# Supplementary material for: Genome-wide allele-specific expression in multi-tissue samples from healthy male baboons reveals the transcriptional complexity of mammals
Source: Cell Genom. 2025 Apr 4;5(5):100823. doi: 10.1016/j.xgen.2025.100823 (PMC12143330; doi:10.1016/j.xgen.2025.100823)
Supplement: Document S1. Figures S1–S6 [file mmc1.pdf]

Cell Genomics, Volume *5*

Supplemental information

Genome-wide allele-specific expression

in multi-tissue samples from healthy male baboons

reveals the transcriptional complexity of mammals

Ramesh Ramasamy, Muthuswamy Raveendran, R. Alan Harris, Hiep D. Le, Ludovic S. Mure, Giorgia Benegiamo, Ouria Dkhissi-Benyahya, Howard Cooper, Jeffrey Rogers, and Satchidananda Panda

1. Empirical null hypothesis

The assumed null hypothesis for our analysis was an allelic ratio of 0.5, anticipating that half of the mapped reads would carry the reference allele and the other half the alternate allele. Any statistically significant deviation from this ratio was considered indicative of allele-specific expression (ASE). However, various technical confounders, including read mapping bias, can skew this assumption. Reads carrying the alternate allele inherently possess at least one more mismatch compared to those carrying the reference allele, potentially reducing their chances of mapping correctly. To mitigate this potential bias, we enabled the WASP mode in STAR mapping using the "–waspOutputMode" flag. Subsequently, we retained only those reads specifically marked with the "vW:i:1" flag. We then computed an empirical null hypothesis by comparing the number of reads carrying the reference allele versus the alternate allele across all heterozygous loci within each sample.

For our analysis, we focused exclusively on heterozygous SNVs located within the transcribed regions of protein-coding genes, requiring a minimum of ten RNA-Seq reads mapped to each SNV. To evaluate potential mapping bias, we calculated the reference bias for each individual SNV by dividing the number of reads carrying the reference allele by the total number of reads mapped. Across all 125 samples and all SNVs examined, the median reference bias was 0.5 (**Figure SD1**), suggesting that mapping bias, if present, was effectively controlled. At the individual sample level, the median reference bias remained at 0.5 in all except pancreatic samples (see the table below).

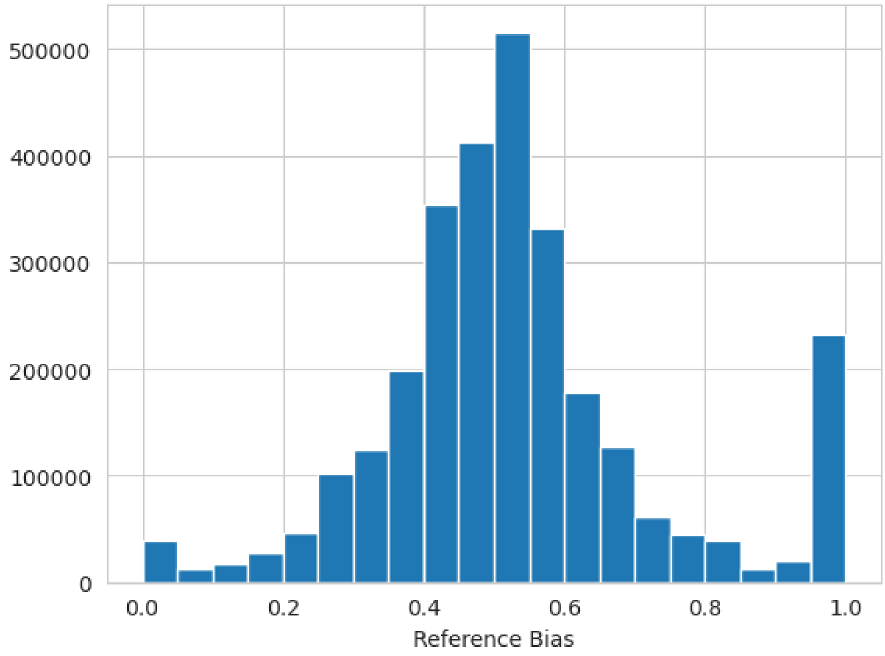

Figure SD1: Histogram illustrating the distribution of reference bias across all SNVs and samples.

Animal	Tissue	Median Reference Bias
Baboon-1	HEA	0.5000
Baboon-1	HIP	0.5000
Baboon-1	ILE	0.5000
Baboon-1	LUN	0.5000
Baboon-1	MUG	0.5000
Baboon-1	PAN	0.5217
Baboon-1	SPL	0.5000
Baboon-1	THA	0.5000
Baboon-1	VIC	0.5000
Baboon-1	WAT	0.5021
Baboon-2	HEA	0.5034
Baboon-2	HIP	0.5000
Baboon-2	ILE	0.5000
Baboon-2	KIC	0.5000
Baboon-2	LUN	0.5000
Baboon-2	MUG	0.5067
Baboon-2	PAN	0.5172
Baboon-2	SPL	0.5000
Baboon-2	THA	0.5000
Baboon-2	VIC	0.5000
Baboon-2	WAT	0.5049
Baboon-3	HEA	0.5052
Baboon-3	HIP	0.5000
Baboon-3	ILE	0.5000
Baboon-3	KIC	0.5000
Baboon-3	LUN	0.5000
Baboon-3	MUG	0.5000
Baboon-3	PAN	0.5041
Baboon-3	SPL	0.5050
Baboon-3	THA	0.5000
Baboon-3	VIC	0.5000
Baboon-4	HEA	0.5000
Baboon-4	HIP	0.5000
Baboon-4	ILE	0.5000
Baboon-4	KIC	0.5000
Baboon-4	MUG	0.5027
Baboon-4	PAN	0.5143
Baboon-4	SPL	0.5055

Baboon-4	THA	0.5000
Baboon-4	VIC	0.5000
Baboon-5	HEA	0.5000
Baboon-5	HIP	0.5000
Baboon-5	ILE	0.5000
Baboon-5	KIC	0.5000
Baboon-5	LUN	0.5000
Baboon-5	MUG	0.5091
Baboon-5	PAN	0.5097
Baboon-5	SPL	0.5056
Baboon-5	THA	0.5000
Baboon-5	VIC	0.5000
Baboon-5	WAT	0.5049
Baboon-6	HEA	0.5000
Baboon-6	HIP	0.5000
Baboon-6	ILE	0.5000
Baboon-6	KIC	0.5000
Baboon-6	LUN	0.5000
Baboon-6	SPL	0.5000
Baboon-6	THA	0.5000
Baboon-6	VIC	0.5000
Baboon-6	WAT	0.5000
Baboon-7	HEA	0.5000
Baboon-7	HIP	0.5000
Baboon-7	ILE	0.5000
Baboon-7	KIC	0.5000
Baboon-7	LUN	0.5000
Baboon-7	MUG	0.5030
Baboon-7	PAN	0.5082
Baboon-7	SPL	0.5000
Baboon-7	THA	0.5000
Baboon-7	VIC	0.5000
Baboon-7	WAT	0.5000
Baboon-8	HEA	0.5000
Baboon-8	HIP	0.5000
Baboon-8	ILE	0.5021
Baboon-8	KIC	0.5000
Baboon-8	LUN	0.5000
Baboon-8	MUG	0.5000
Baboon-8	PAN	0.5227
Baboon-8	SPL	0.5000

Baboon-8	THA	0.5000
Baboon-8	VIC	0.5000
Baboon-8	WAT	0.5033
Baboon-9	HEA	0.5000
Baboon-9	HIP	0.5000
Baboon-9	ILE	0.5000
Baboon-9	KIC	0.5000
Baboon-9	LUN	0.5000
Baboon-9	MUG	0.5000
Baboon-9	PAN	0.5000
Baboon-9	SPL	0.5000
Baboon-9	THA	0.5000
Baboon-9	VIC	0.5000
Baboon-9	WAT	0.5000
Baboon-10	HEA	0.5000
Baboon-10	HIP	0.5000
Baboon-10	ILE	0.5000
Baboon-10	KIC	0.5000
Baboon-10	MUG	0.5000
Baboon-10	PAN	0.5039
Baboon-10	SPL	0.5016
Baboon-10	THA	0.5000
Baboon-10	VIC	0.5000
Baboon-10	WAT	0.5000
Baboon-11	HEA	0.5000
Baboon-11	HIP	0.5000
Baboon-11	ILE	0.5000
Baboon-11	KIC	0.5000
Baboon-11	LUN	0.5000
Baboon-11	MUG	0.5000
Baboon-11	PAN	0.5152
Baboon-11	SPL	0.5000
Baboon-11	THA	0.5000
Baboon-11	VIC	0.5000
Baboon-11	WAT	0.5085
Baboon-12	HEA	0.5000
Baboon-12	HIP	0.5000
Baboon-12	ILE	0.5000
Baboon-12	KIC	0.5000
Baboon-12	LUN	0.5000
Baboon-12	MUG	0.5000

Baboon-12	PAN	0.5060
Baboon-12	SPL	0.5000
Baboon-12	THA	0.5000
Baboon-12	VIC	0.5000
Baboon-12	WAT	0.5006

Table SD1: Median reference bias across all assayed SNVs in a sample.

2. Empirical dispersion estimation

Overdispersion significantly impacts ASE estimation, affecting both independent beta-binomial tests and MBASED analysis. To quantify the dispersion in our data, we selected heterozygous SNVs within transcribed regions of protein-coding genes, each supported by at least ten RNA-Seq reads. To ensure robust dispersion estimation, we focused on biallelic SNVs expressed in at least five samples. This criterion ensured that the selected SNVs expressed both reference and alternate alleles in at least one of the 125 analyzed samples. We employed the log-likelihood method of the beta-binomial distribution, implemented in the Python SciPy package, to calculate the dispersion within our dataset. Based on the resulting dispersion distribution, we applied a uniform dispersion value of 0.001 for both MBASED and the beta-binomial test.

Decile	0.1	0.2	0.3	0.4	0.5	0.6	0.7	0.8	0.9
Dispersion	0.000079	0.00026	0.00045	0.00062	0.00078	0.00093	0.0011	0.0013	0.0019

Table SD2: Distribution of empirically estimated dispersion.

3. Power of beta-binomial test

We hypothesized that the power of the beta-binomial test to identify ASE would be largely influenced by both the effect size and the number of mapped reads. To investigate this relationship, we simulated datasets with varying allelic proportions, representing effect sizes from 0.01 to 0.99, and read depths of 200, 100, 75, 50, 25, and 10 reads. These read depths represent the total number of reads aligned to a given locus. Our simulations were designed to closely reflect the empirical read depths observed in our data (**Table SD3**), as well as the range of effect sizes identified (**Figure SD1**). The simulations were conducted using a dispersion parameter (ρ) of 0.001. We generated 10,000 datasets for each combination of effect size and read depth, simulating data under the alternative hypothesis of allelic imbalance. Power was

then calculated as the proportion of these datasets where the beta-binomial test successfully rejected the null hypothesis at a significance level of 0.05. We computed p-values using the cumulative distribution function (CDF) of the beta-binomial distribution. Given the two-tailed nature of the test, the p-value was determined as twice the minimum value between the CDF value and its complement.

Decile	0.1	0.2	0.3	0.4	0.5	0.6	0.7	0.8	0.9
#reads mapped	12	16	20	26	34	46	64	96	175

Table SD3: Distribution of number of reads mapped to a given locus.

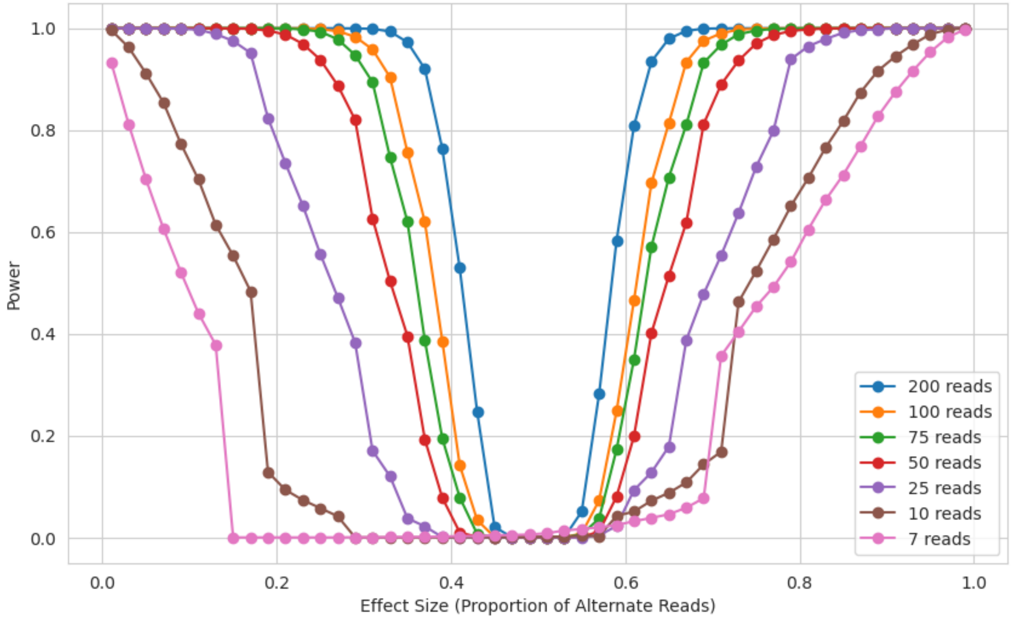

Figure SD3.1: Influence of effect sizes and read depth in successful ASE estimation after incorporating multiple tests.

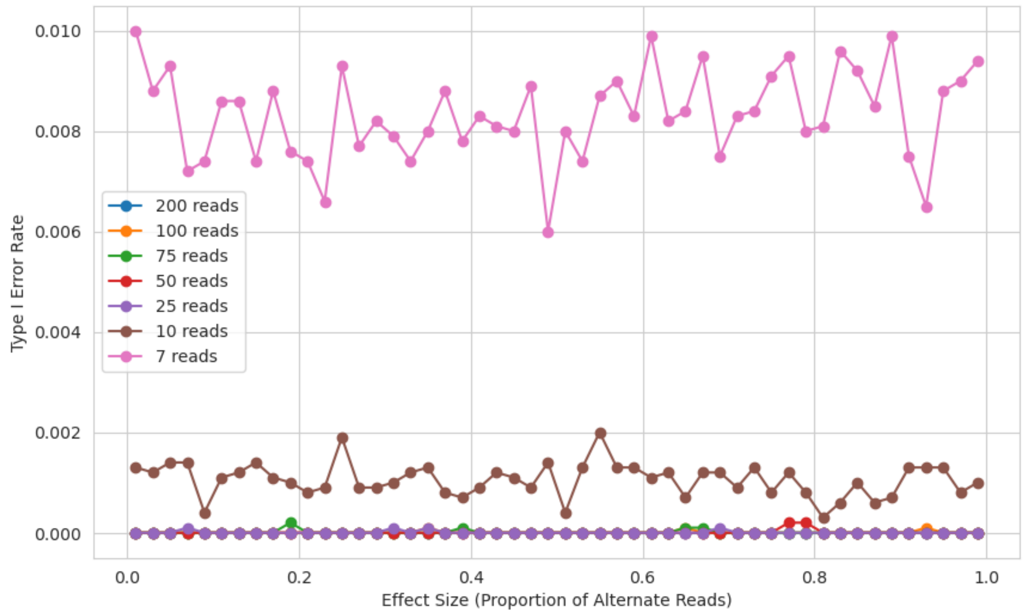

Figure SD3.2: Influence of effect sizes and read depth in Type-I error estimation.

4. Concordance between MBASED and SNV level beta-binomial test

MBASED employs meta-analysis, aggregating information across multiple SNVs to detect ASE at the gene level. In addition to MBASED, we used the beta-binomial test to identify SNV-level ASE, which is essential for understanding the specific effects of individual SNVs. We anticipated a certain degree of concordance between these two tests. Of the 730,673 genes assayed across 125 samples, 74.2% were not significant in either test. Conversely, 13.2% were deemed significant by both tests. Interestingly, 7.5% were significant only in MBASED, while 4.8% reached significance only in the beta-binomial test. It is important to note that a gene was classified as significant in the beta-binomial test if at least one heterozygous SNV located within that gene showed significance.

To gain a deeper understanding of the observed concordance and discrepancies between these two tests, we calculated and compared various metrics.

Deciles	0.1	0.2	0.3	0.4	0.5	0.6	0.7	0.8	0.9
Both	0.000	0.000	0.000	0.000	0.000	0.000	0.001	0.006	0.020
Neither	0.155	0.278	0.405	0.524	0.636	0.750	0.832	0.925	0.996
Beta Binom	0.078	0.118	0.174	0.249	0.346	0.465	0.602	0.757	0.909
MBASED	0.000	0.000	0.002	0.005	0.010	0.016	0.023	0.031	0.041

Table SD4.1: Distribution of MBASED P values across three groups.

Deciles	0.1	0.2	0.3	0.4	0.5	0.6	0.7	0.8	0.9
Both	0.000	0.000	0.000	0.000	0.001	0.004	0.008	0.019	0.033
Neither	0.164	0.263	0.379	0.493	0.591	0.725	0.837	0.931	1.000
Beta Binom	0.000	0.003	0.007	0.011	0.018	0.024	0.031	0.040	0.046
MBASED	0.069	0.084	0.111	0.147	0.173	0.230	0.278	0.401	0.630

Table SD4.2: Distribution of beta binomial P values across three groups.

Deciles	0.1	0.2	0.3	0.4	0.5	0.6	0.7	0.8	0.9
Both	0.15	0.25	0.33	0.50	0.56	0.80	1.00	1.00	1.00
Neither	0.00	0.00	0.00	0.00	0.00	0.00	0.00	0.00	0.00
Beta Binom	0.09	0.13	0.14	0.18	0.22	0.25	0.33	0.50	0.50
MBASED	0.00	0.00	0.00	0.00	0.00	0.00	0.00	0.00	0.00

Table SD4.3: Proportion of SNVs with beta-binomial p values < 0.05.

Deciles	0.1	0.2	0.3	0.4	0.5	0.6	0.7	0.8	0.9
Both	1	2	3	4	5	6	8	11	16
Neither	1	1	1	1	2	2	3	4	6
Beta Binom	2	3	4	4	5	7	8	10	14
MBASED	1	2	2	3	3	4	5	7	9

Table SD4.4: Number of SNVs in a gene

Deciles	0.1	0.2	0.3	0.4	0.5	0.6	0.7	0.8	0.9
Both	15	20	26	34.5	46	64	92.5	148	304
Neither	13	16	20	25	32	43	58.5	86	152
Beta Binom	17	22	27.5	35	44.5	57.5	77	111	195
MBASED	14	17	22	27	35	46	63.5	95	170

Table SD4.5: Total read number of reads

Deciles	0.1	0.2	0.3	0.4	0.5	0.6	0.7	0.8	0.9
Both	0.000	0.000	0.000	0.002	0.019	0.085	0.245	0.585	0.991
Neither	0.024	0.085	0.172	0.279	0.403	0.539	0.681	0.826	0.959
Beta Binom	0.000	0.000	0.000	0.001	0.003	0.008	0.017	0.038	0.091
MBASED	0.000	0.015	0.092	0.228	0.384	0.548	0.709	0.864	0.985

Table SD4.6: Median heterogeneity among SNVs in a gene identified by MBASED.

MBASED demonstrates increased power to detect subtle ASE, especially in genes with a greater number of SNVs, even when the allelic imbalance is not significant at every individual SNV. This is reflected in a higher proportion of significant SNVs and lower MBASED p-values in genes significant only by MBASED. Notably, MBASED appears tolerant of heterogeneity in ASE signals among SNVs within a gene, as evidenced by higher heterogeneity p-values in genes uniquely identified by MBASED. Conversely, genes significant only in the beta-binomial analysis often exhibit a more mixed heterogeneity pattern, potentially explaining why MBASED might not flag them as having consistent gene-level ASE. These findings underscore the importance of considering both the number of informative SNVs, the consistency of the ASE signal across those SNVs, and the inherent differences in the statistical frameworks of the two methods when interpreting results.

5. **Highly expressed genes more often show ASE**

We examined the relationship between gene expression levels (TPM) and ASE using a mixed-effects logistic regression model. Using gene expression (TPM) as a fixed effect and individual

sample names as a random effect, the analysis revealed that with a rise in gene expression, there is an increase in the log odds (with a coefficient of 1.96) of a gene exhibiting ASE. This aligns with the prior reports indicating that highly expressed genes more frequently exhibit ASE, presumably due to greater selection pressures. Genes with higher expression levels have been shown to be more efficient at suppressing the expression of deleterious alleles.
