# Supplementary material for: Genome-wide allele-specific expression in multi-tissue samples from healthy male baboons reveals the transcriptional complexity of mammals
Source: Cell Genom. 2025 Apr 4;5(5):100823. doi: 10.1016/j.xgen.2025.100823 (PMC12143330; doi:10.1016/j.xgen.2025.100823)
Supplement: Document S2. Supplementary data [file mmc2.pdf]

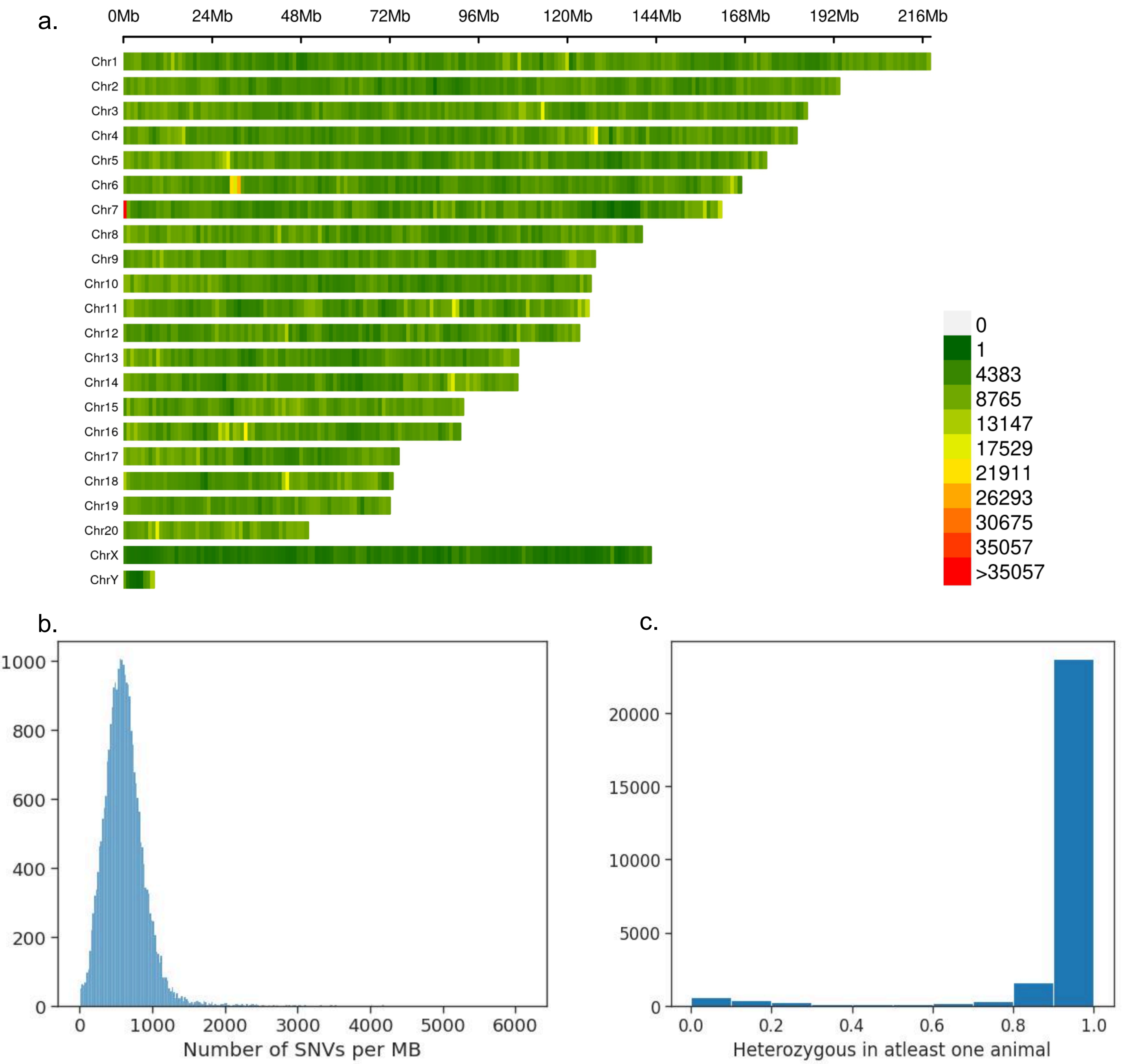

Figure S1: a, An ideogram illustrates the density of 16 million SNVs identified across 12 baboon genomes in the study. A non-overlapping 1 Mb window was utilized to compute the SNV density. **b**, A histogram illustrates the distribution of the number of SNVs discovered in a non-overlapping 1 MB window. **c**, A majority of the SNVs identified in these non-overlapping 1 Mb windows were found to be heterozygous in at least one animal. Related to Figure 1.

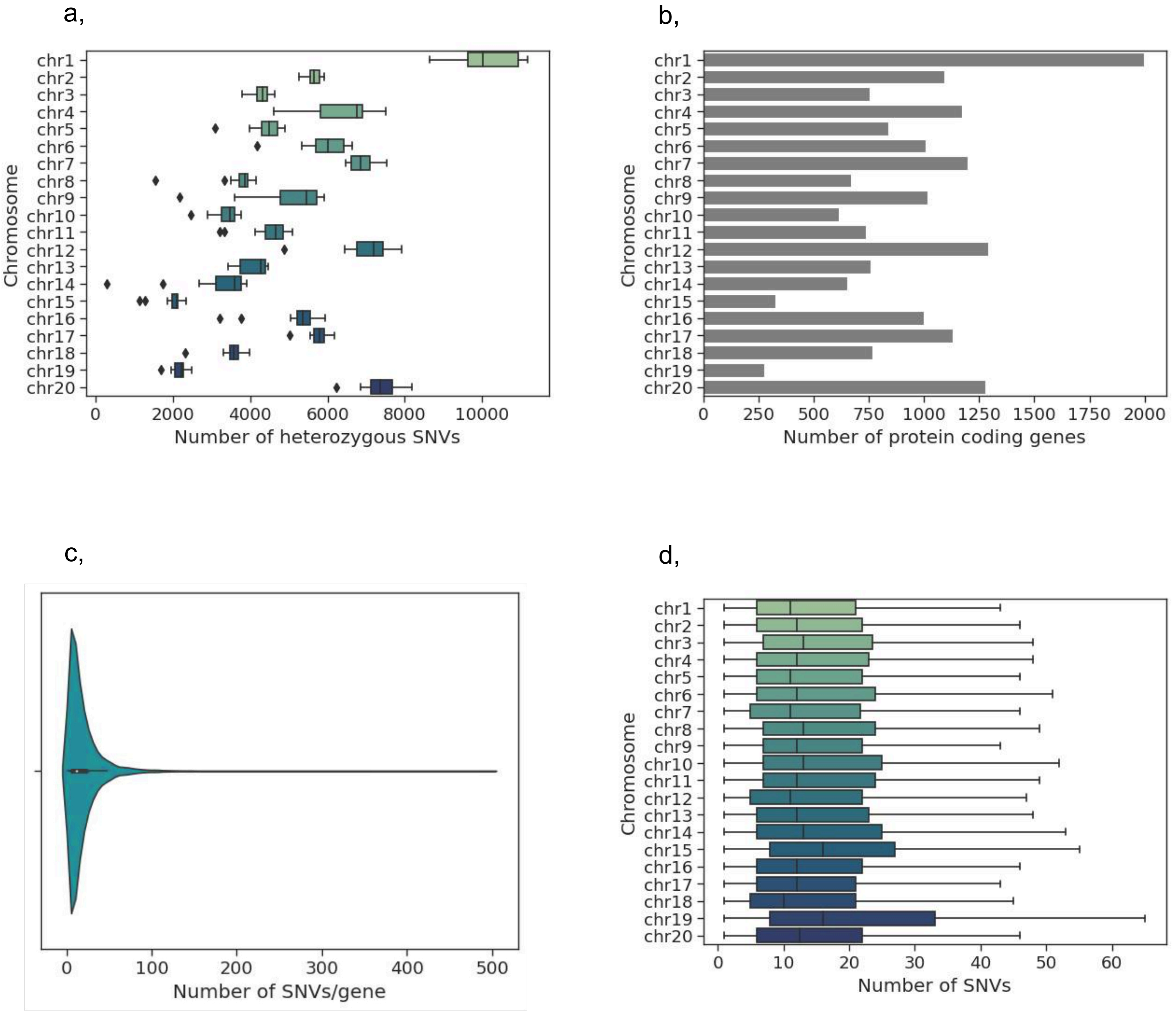

Figure S2: a, Boxplots illustrate the number of heterozygous SNVs located in coding and UTR regions across the 20 autosomes of baboons, revealing a proportionality to the number of protein-coding genes annotated on each chromosome. **b,** The number of protein-coding genes annotated on a chromosome does not always correlate with the chromosome's size. **c**, A median of 12 heterozygous SNVs were identified in the coding and UTR regions of a protein-coding gene. **d**, The density of heterozygous SNVs found within protein-coding genes was largely consistent across the 20 autosomes**.** Related to Figure 2.

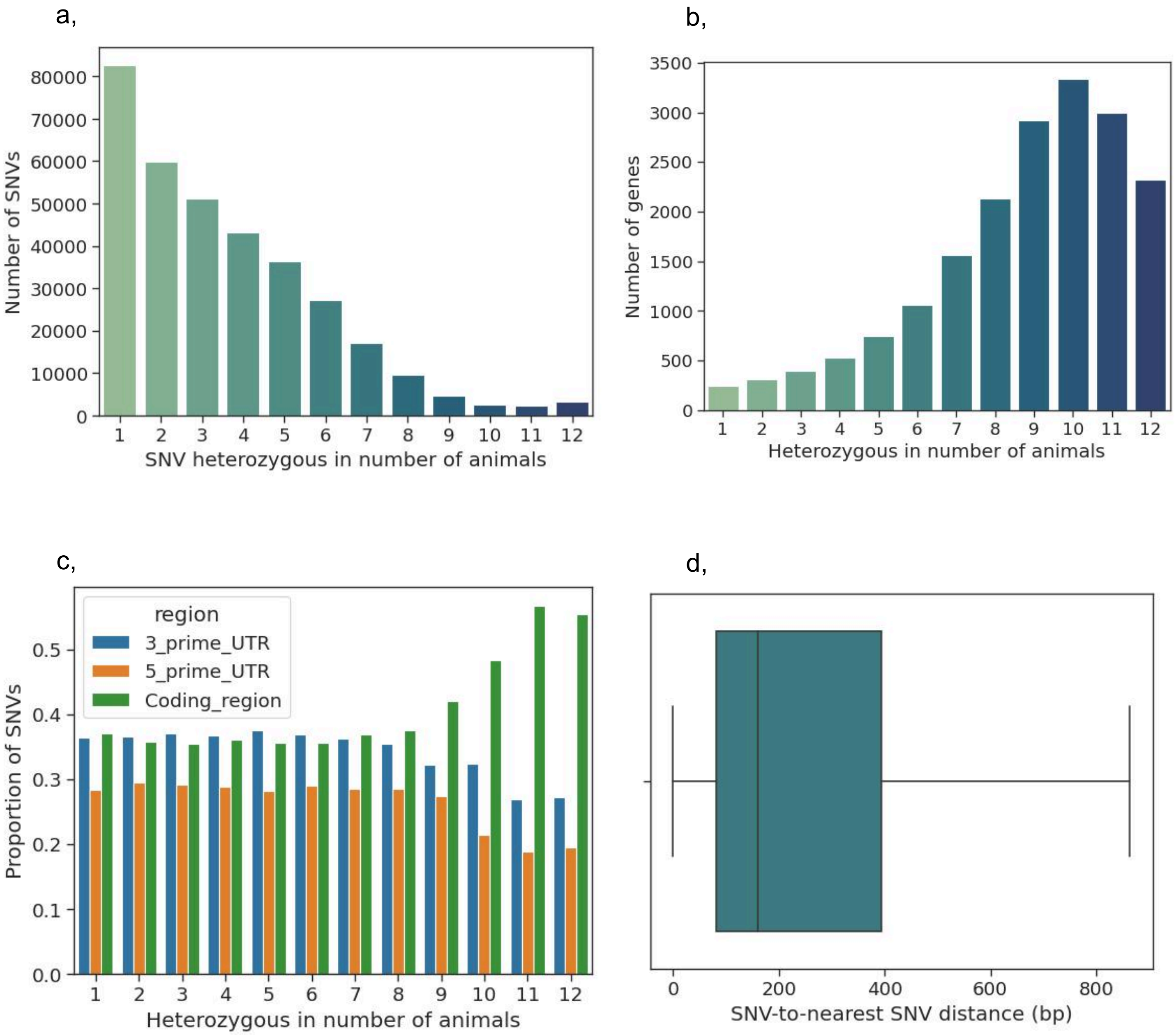

Figure S3: **a**, A bar plot illustrates the number of SNVs that are heterozygous in a varying number of animals. For this analysis, SNVs that were heterozygous in at least one animal and located in the coding and UTR regions of protein-coding genes were considered. **b**, The majority of protein-coding genes contained at least one heterozygous SNV, making these genes assayable in the majority of the baboons studied. **c**, SNVs located in coding regions were more likely to be heterozygous across multiple animals. However, it is important to note that SNVs found to be heterozygous across all 12 baboons were significantly fewer than those found to be heterozygous in a smaller number of animals. **d**, In the transcribed regions of protein-coding genes, one heterozygous SNV was found at a median frequency of every 161 base pairs (bp). Related to Figure 3.

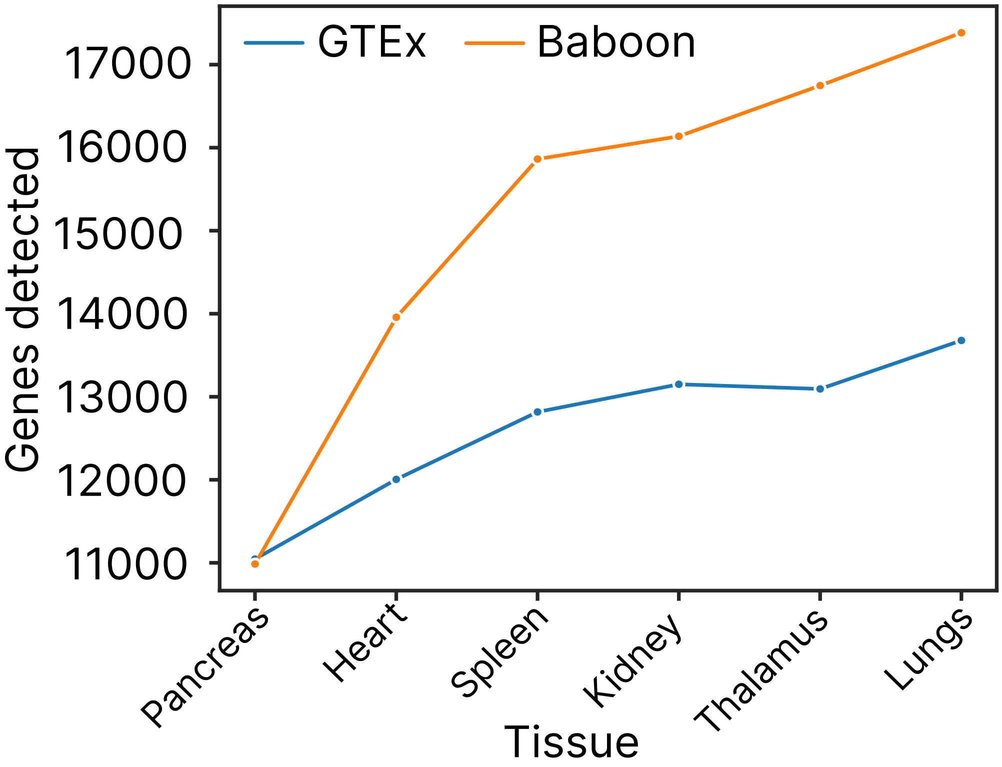

Figure S4. Number of genes expressed in humans vs baboons. Related to Figure 3.

a,

b,

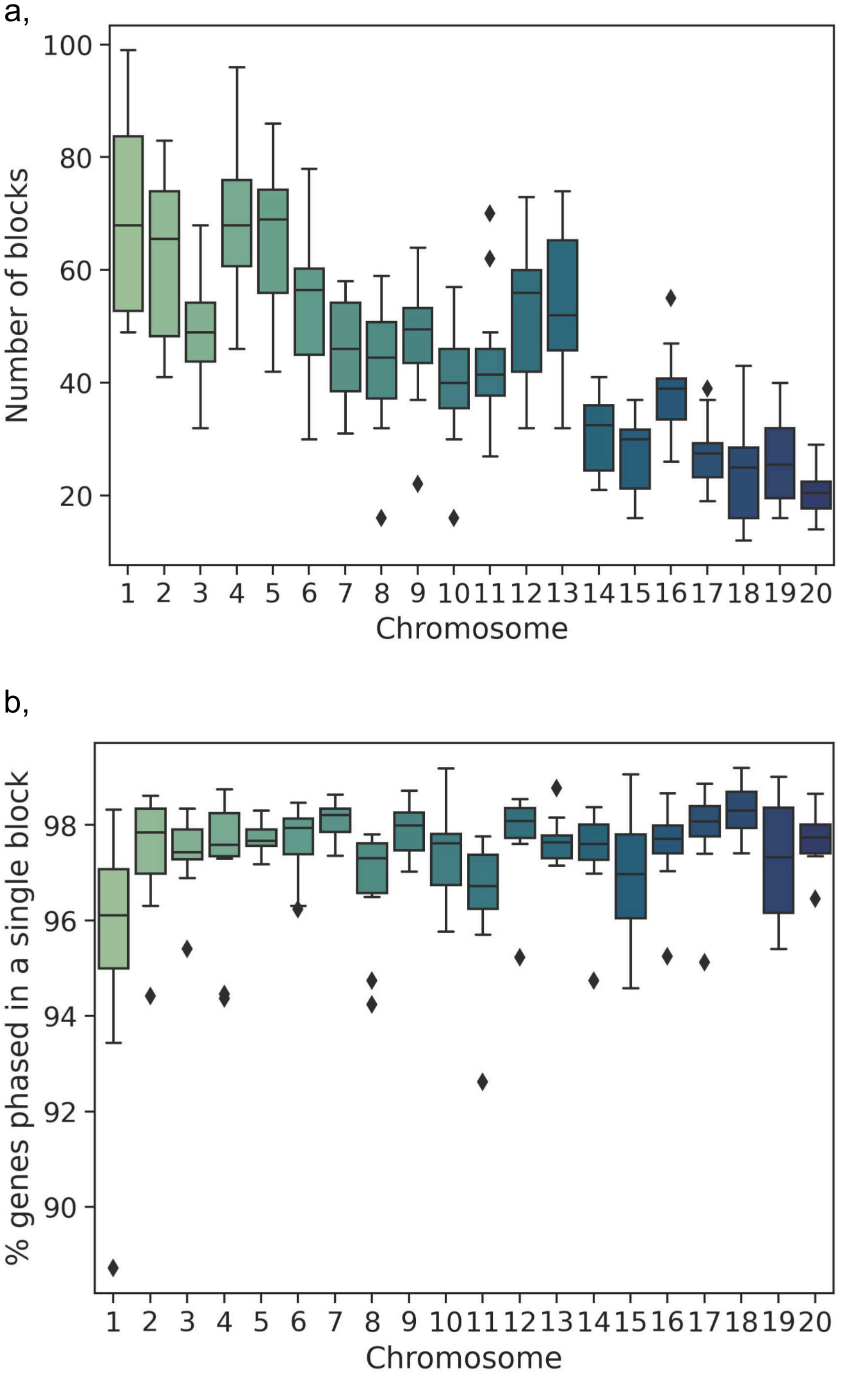

Figure S5: **a**, A boxplot illustrates that 90% of SNVs were phased into a number of blocks, with shorter chromosomes like chromosome 20 being phased into fewer blocks compared to longer chromosomes such as chromosome 1. This analysis includes all heterozygous SNVs identified in each chromosome, and the boxplot represents the variance observed across the genomes of the 12 baboons. **b**, In terms of the percentage of genes, 100% of SNVs were phased as a single block. Across all 20 chromosomes, 100% of SNVs located on almost all of the protein-coding genes were phased as a single block, indicating a high level of phasing efficiency. Related to Figure 3.

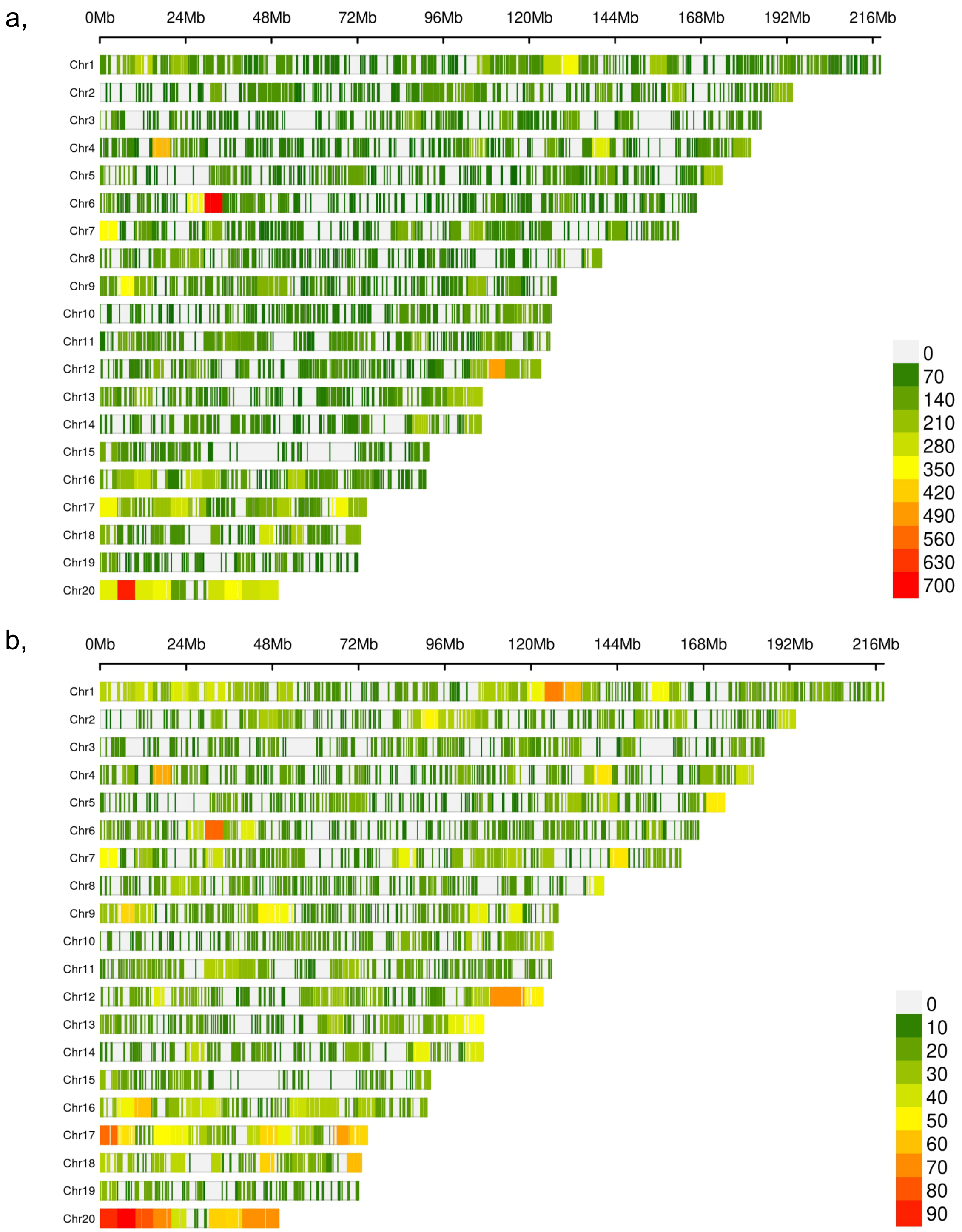

Figure S6. a, Ideogram illustrating the density of SNVs with ASE traits across 20 autosomes. **b**, Ideogram illustrating the density of ASE genes across 20 autosomes. Related to Figure 4.
