# Supplementary material for: Genome-wide allele-specific expression in multi-tissue samples from healthy male baboons reveals the transcriptional complexity of mammals
Source: Cell Genom. 2025 Apr 4;5(5):100823. doi: 10.1016/j.xgen.2025.100823 (PMC12143330; doi:10.1016/j.xgen.2025.100823)
Supplement: Document S3. Article plus supplemental information [file mmc18.pdf]

Cell Genomics

Article

Genome-wide allele-specific expression in multi-tissue samples from healthy male baboons reveals the transcriptional complexity of mammals

Graphical abstract

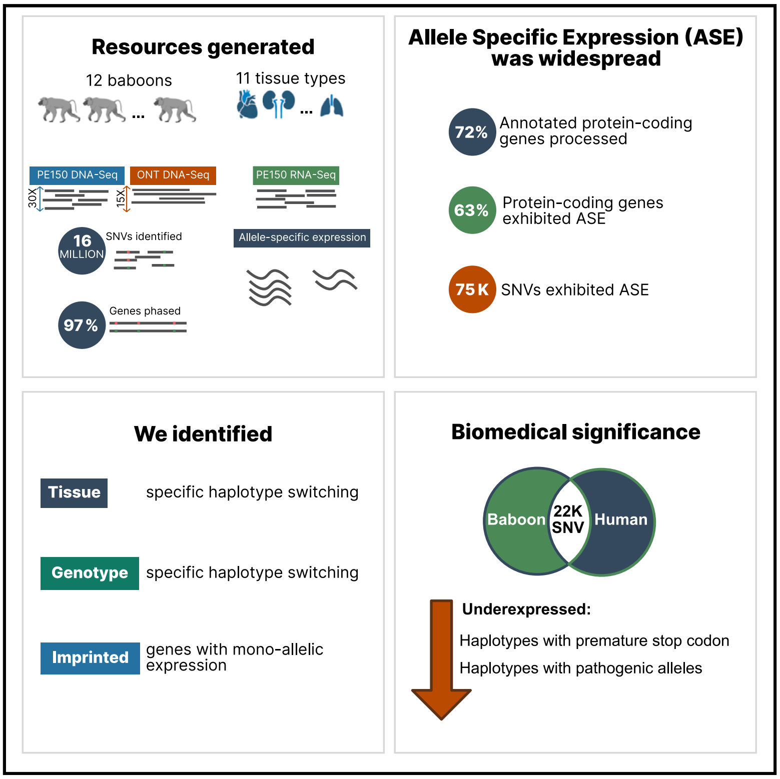

Highlights

- Baboons are ∼2× more heterozygous than humans, enabling extensive ASE analysis
- Over 63% of protein-coding genes exhibited ASE in at least one sample
- We identified ASE variants that also exist in the human population
- Haplotypes with pathogenic alleles were actively underexpressed

Authors

Ramesh Ramasamy, Muthuswamy Raveendran, R. Alan Harris, ..., Howard Cooper, Jeffrey Rogers, Satchidananda Panda

Correspondence

jr13@bcm.edu (J.R.), satchin@salk.edu (S.P.)

In brief

Knowing which parental allele is preferentially expressed or suppressed in our genome has profound implications. Using tissues from healthy, young baboons, Ramasamy et al. found widespread allele-specific expression in protein-coding genes. A subset of the corresponding underexpressed variants is classified as pathogenic and is also present in humans.

Ramasamy et al., 2025, Cell Genomics 5, 100823
May 14, 2025 © 2025 The Author(s). Published by Elsevier Inc.
https://doi.org/10.1016/j.xgen.2025.100823

CellPress

Cell Genomics

CellPress OPEN ACCESS

Article

Genome-wide allele-specific expression in multi-tissue samples from healthy male baboons reveals the transcriptional complexity of mammals

Ramesh Ramasamy,[1] Muthuswamy Raveendran,[2] R. Alan Harris,[2] Hiep D. Le,[1] Ludovic S. Mure,[1,5] Giorgia Benegiamo,[3] Ouria Dkhissi-Benyahya,[4] Howard Cooper,[4] Jeffrey Rogers,[2,*] and Satchidananda Panda[1,6,*]

[1]Salk Institute for Biological Studies, 10010 North Torrey Pines Road, La Jolla, CA 92037, USA

[2]Human Genome Sequencing Center and Department of Molecular and Human Genetics, Baylor College of Medicine, Houston, TX 77030, USA

[3]Laboratory of Integrative Systems Physiology, Institute of Bioengineering, School of Life Sciences, Ecole Polytechnique Fédérale de Lausanne, 1015 Lausanne, Switzerland

[4]Univ Lyon, Université Claude Bernard Lyon 1, INSERM, Stem Cell and Brain Research Institute U1208, 69500 Bron, France

[5]Present address: Inselspital, Department of Ophthalmology, Bern, Switzerland

[6]Lead contact

*Correspondence: jr13@bcm.edu (J.R.), satchin@salk.edu (S.P.)

https://doi.org/10.1016/j.xgen.2025.100823

SUMMARY

Allele-specific expression (ASE) is pivotal in understanding the genetic underpinnings of phenotypic variation within species, differences in disease susceptibility, and responses to environmental factors. We processed 11 different tissue types collected from 12 age-matched healthy olive baboons (*Papio anubis*) for genome-wide ASE analysis. By sequencing their genomes at a minimum depth of 30×, we identified over 16 million single-nucleotide variants (SNVs). We also generated long-read sequencing data, enabling the phasing of all variants present within the coding regions of 96.5% of assayable protein-coding genes as a single haplotype block. Given the extensive heterozygosity of baboons relative to humans, we could quantify ASE across 72% of the total annotated protein-coding gene set. We identified genes that exhibit ASE and affect specific tissues and genotypes. We discovered ASE SNVs that also exist in human populations with identical alleles and that are designated as pathogenic by both the PrimateAI-3D and AlphaMissense models.

INTRODUCTION

Advances in human genetics continue to uncover genetic variations linked to a wide spectrum of human diseases.[1,2] Genome-wide association studies (GWASs) using large cohorts have significantly advanced the mapping of variants correlated with the risk of complex disorders, including coronary heart disease,[3,4] hypertension,[5] schizophrenia,[6] and others.[7] These studies reveal that many strongly associated genetic variants reside outside protein-coding regions, suggesting that they influence disease risk by modifying gene expression. Current evidence indicates that such expression quantitative trait loci (eQTLs) account for a considerable portion of the variation in risk for common complex diseases.[8] However, substantial challenges hinder the definitive identification of individual causal disease variants, even after GWAS findings have highlighted significant associations. Furthermore, quantitative gene expression levels often vary across tissues, and the two alleles of a gene within a given genome frequently exhibit imbalanced expression levels in one or more tissues.[9] This phenomenon, known as allele-specific expression (ASE), can impact the penetrance of pathogenic alleles, impede the ability of association analyses to identify causal variants, and stem from eQTLs, imprinting, or other cellular mechanisms such as tissue-specific epigenetic modifications.[10,11] Consequently, the complex interplay between positive GWAS tests, eQTLs, tissue-specific expression levels, and their ultimate contribution to disease risk remains poorly understood.[12]

ASE exerts a significant influence on a multitude of biological processes. Studies have linked ASE to the risk of Crohn disease,[13] cancer development,[14] aging rates,[15] susceptibility to cardiac diseases,[16] and complex phenotypes in other outbred mammals.[17] This mechanism acts as a crucial intermediary step, modulating the dosage of specific haplotypes and consequently impacting phenotypic expression. The broad significance of ASE stems from the presence of 2–2.8 million heterozygous variants within individual human genomes.[18,19] Therefore, it is unsurprising that variants associated with specific genetic diseases and disorders frequently exist in a heterozygous state.[3,20] Furthermore, recent research suggests that ASE can lead to the underexpression of harmful alleles, potentially contributing to the variable penetrance of detrimental phenotypes or enabling these variants to circumvent negative selection.[10] Detailed characterization of the prevalence of ASE and identification of the affected

This is an open access article under the CC BY license (http://creativecommons.org/licenses/by/4.0/).

genes and tissues will not only deepen our understanding of genotype-phenotype correlations but also broaden our knowledge of human genome function, particularly the clinically relevant genetic mechanisms underlying diseases.

By analyzing RNA-sequencing (RNA-seq) reads carrying single-nucleotide variant (SNV) alleles, it is possible to assign reads to specific haplotypes and alleles, and to examine the expression of individual homologs.[21] Previous multi-tissue ASE studies in humans[9,10,22,23] have relied primarily on tissues harvested from deceased individuals at varying ages, and many of the subjects suffered from various health conditions. While these studies have offered a critically important glimpse into the intricate landscape of ASE in humans, gene expression could be influenced by age and health states.[24] Given the challenges of obtaining suitable tissue samples from healthy humans, nonhuman primates such as baboons (genus *Papio*) serve as excellent models for studying the ASE of genes implicated in various genetic diseases and disorders.[25,26] With a 94% genome-wide similarity to humans[27] and a nearly 3-fold increase in heterozygosity within protein-coding segments,[28] baboons provide a valuable resource for these investigations. A recent study has highlighted the value of baboons for eQTL analysis of gene × environment interactions that influence risk for cardiovascular disease.[29] To develop a better understanding of quantitative gene expression and ASE, with the long-term goal of improving our ability to delineate genetic risk factors for human diseases that are influenced by this complex process, we undertook an extensive analysis of genome-wide ASE in 12 male olive baboons (*P. anubis*) across 11 different tissue types. We demonstrate that the increased heterozygosity in this nonhuman primate genome not only facilitates the analysis of a larger number of protein-coding genes but also offers the opportunity to examine the ASE of variants found in both baboon and human genomes. Furthermore, we underscore the value of acquiring tissue specimens from healthy baboons of equivalent young adult age. This methodology, while not feasible in human subjects, is paramount for the unbiased examination of ASE and its broad implications for disease. Our findings culminate in the identification of a significant number of ASE genes that have clinical relevance, offering a promising foundation for subsequent application to human biomedical research.

RESULTS

Dataset overview

In this study, we utilized 11 different tissue sample types, including from the brain, heart, lungs, kidney, gastrointestinal tract, muscle, and adipose tissue (see Figure 1A for the complete list), from our previous study,[30] materials collected from each of the 12 male olive baboons (Table S1). These baboons, aged between 5 and 6 years and weighing between 7 and 11 kg, were screened for the absence of any infectious diseases and kept under controlled conditions for 1 month before tissue collection. Prior multi-tissue transcriptome studies had not found any genomic signatures of disease.[30] Genomic DNA isolated from each of the 12 baboons was sequenced using Illumina NovaSeq methods and Oxford Nanopore Technologies (ONT) long-reads technology, achieving a minimum sequencing depth of 30× and 15×, respectively (Table S2). We used Illumina 2 × 150 bp paired reads primarily for variant calling (Figure 1B). As the main purpose of the study was to study ASE events at a gene level, it was necessary to place variants into distinct haplotype blocks. Therefore, we utilized both Illumina PE150 and ONT long reads to phase variants into haplotypes. To investigate the patterns of ASE in individual genes, we extracted total RNA from 11 different tissue types obtained from the 12 baboons, resulting in a total of 132 samples. Complementary DNA (cDNA) synthesized from total RNA was sequenced to a median depth of 25 million Illumina read pairs (Table S2). Principal-component analysis (PCA) based on gene expression levels showed distinct tissue-type clustering (Figure 1C), emphasizing the importance of harvesting tissues under controlled conditions from healthy baboons. After excluding samples with fewer than 15 million reads and those that did not correspond to the same tissue type in the PCA plot, we retained 125 samples for further analysis (Table S1).

The baboon genome is at least twice as heterozygous as the typical human genome

We identified 15.7 million SNVs spread across 20 autosomes of baboons, covering a span of 2.3 billion nucleotides[31] (Table S3). This equates to a median variant density of 5 SNVs for every non-overlapping 1-kb segment of the genome (Figures 1D, S1A, and S1B). In an individual baboon genome, between 4.1 and 5.1 million autosomal SNVs were heterozygous, with a median count of 4.8 million (Figures 1E and S1C). The number of SNVs detected and the heterozygosity of our cohort mirrored previous baboon studies.[28,32,33] This implies that the average baboon genome is nearly twice as heterozygous as even the previously reported most heterozygous human genome sets, which typically contain a maximum of 2.8 million heterozygous SNVs.[18] The enhanced heterozygosity of baboon genomes not only expands the set of genes suitable for ASE analysis but it also strengthens the reliability of the ASE calls, given the number of heterozygous variants per individual gene.[11,21] Among the 15.7 million autosomal SNVs detected, 1.5 million were found in the human gnomAD database version 3.1.2[34] with identical alleles, underscoring the potential relevance of our nonhuman primate ASE findings to human biomedical investigations.

We then compared the density of heterozygous SNVs in transcribed regions of protein-coding genes in the baboon genomes with that of humans, using the Human Genome Diversity Project and 1000 Genomes Project callset[34] downloaded from the gnomAD website. An individual baboon genome contained an average of 100,290 heterozygous SNVs in transcribed regions of protein-coding genes, while the human genome contained an average of 36,770 SNVs. This indicates that the exonic regions of baboons are nearly three times more heterozygous than in humans. On average, 13,575 protein-coding genes in a single baboon genome contained heterozygous SNVs, whereas in the human genome, the average was 9,915. Across all 12 baboons we studied, we located 340,210 distinct biallelic heterozygous variants within the transcribed

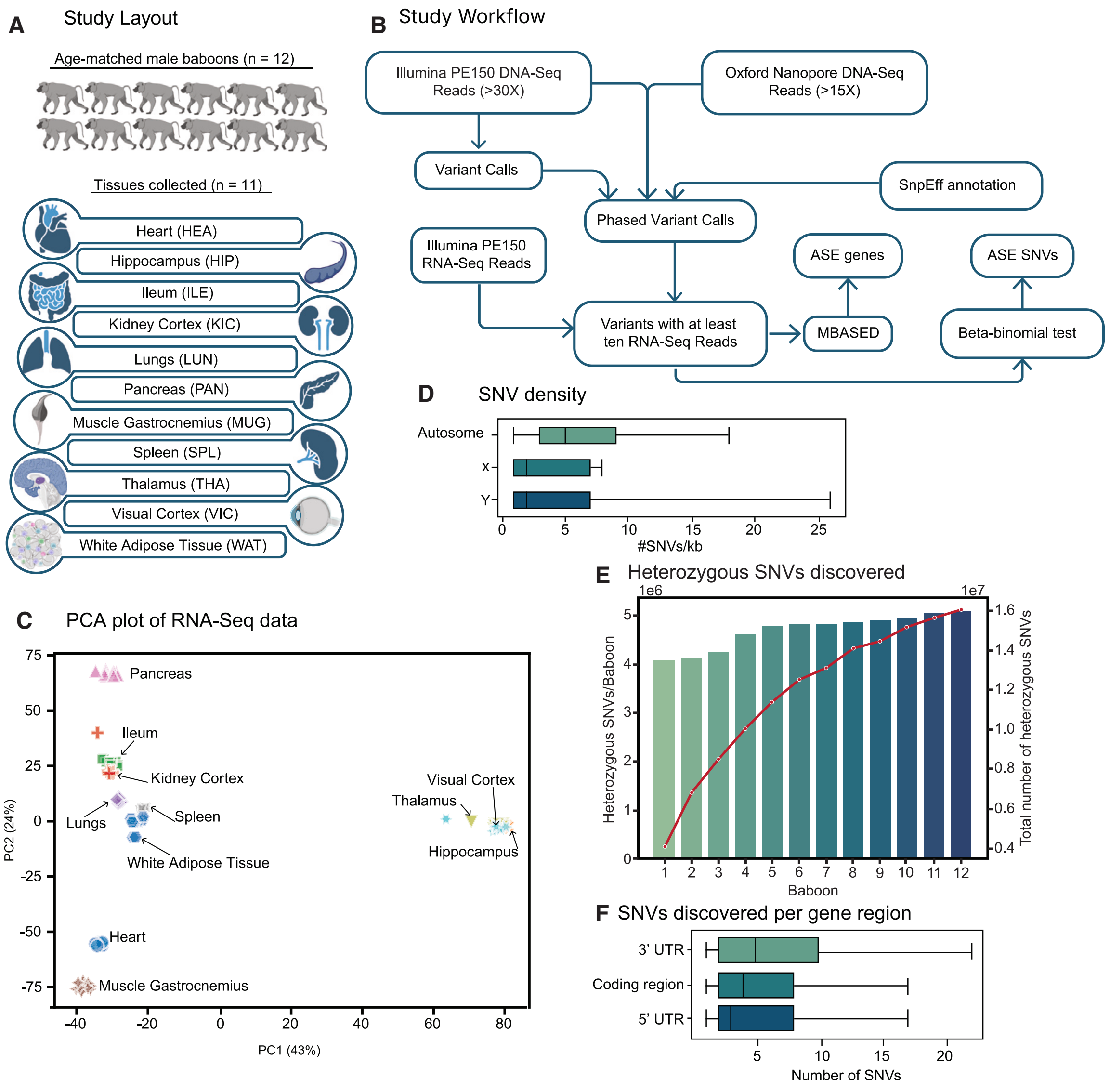

Figure 1. Study design and SNVs discovered

(A) A schematic diagram illustrating the study approach using 12 age-matched male baboons (*Papio anubis*) and the collection of 11 different tissue types.

(B) A schematic diagram illustrating the study workflow in which genomic DNA, sequenced from each baboon, was utilized for variant calling and haplotype phasing, while RNA was employed for ASE analysis.

(C) Principal-component analysis (PCA) of the RNA-seq data from the 11 tissue types from the 12 baboons studied.

(D) Boxplots illustrating the differences in the density of SNVs in autosomes and allosomes. Boxes denote the interquartile range, and horizontal lines denote the median. Whiskers extend to 1.5 times the interquartile range.

(E) Total number of SNVs discovered in our 12-baboon cohort and a median number of SNVs found in a baboon genome.

(F) Density of SNVs within the transcribed regions of protein-coding genes. Boxes denote the interquartile range, and horizontal lines denote the median. Whiskers extend to 1.5 times the interquartile range.

See also Figure S1 and Tables S1, S2, and S3.

regions of 18,585 protein-coding genes (Figure S2A), rendering 88% of the total annotated gene set suitable for ASE analysis, given their expression. We found a median of 12 biallelic heterozygous variants within the transcribed regions of each of these 18,585 genes (Figures 1F and S2B–S2D). Most of these genes exhibited at least one heterozygous SNV in every one of the 12 baboons, making them suitable for ASE analysis across all the baboon samples (Figures S3A–S3D). We restricted our ASE analysis to biallelic variants situated within genes annotated as protein coding in the baboon genome. While other types of RNA, such as long non-coding RNA and microRNA, do play roles in gene expression regulation,[35] their expression patterns were not within the scope of this specific study.

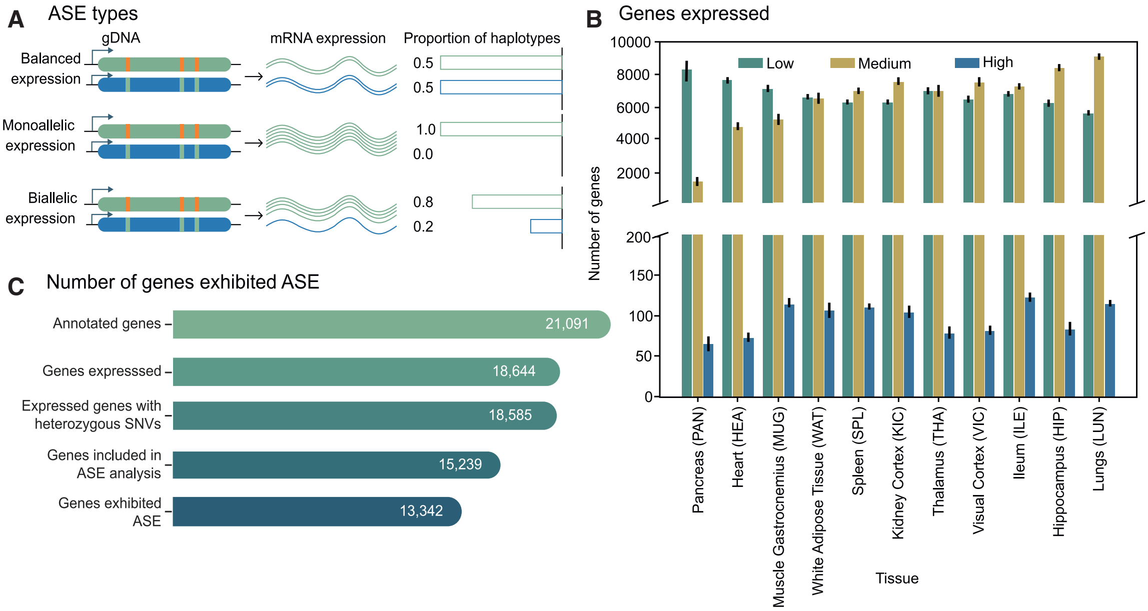

Figure 2. Number of expressed protein-coding genes and those identified as exhibiting ASE

(A) RNA expression was classified as either balanced, monoallelic, or biallelic, based on the proportions of haplotypes in the RNA-seq data.

(B) Number of protein-coding genes with low (between 0.5 and 10 TPM), medium (between 10 and 1,000 TPM), and high (more than 1,000 TPM) levels of gene expression across 11 different tissue types in our 12-baboon cohort.

(C) Over 13,000 protein-coding genes displayed ASE (false discovery rate [FDR] <0.05) in our cohort out of the 15,239 assayed genes. Error bars indicate standard error.

See also Figure S2 and Tables S4 and S5.

The elevated heterozygosity of the baboon genomes enables the use of a large number of protein-coding genes for ASE analysis

For ASE analysis (Figure 2A), heterozygous SNVs that occurred within the transcribed regions and had at least 10 supporting RNA-seq reads were used. These variants are referred to as "informative variants." A detailed discussion on the impact of read depth on power analysis and the type I error rate can be found in Data S1. Protein-coding genes harboring these informative variants are henceforth referred to as "assayable genes." The number of assayable genes available in each sample was largely dependent on the number of genes expressed in that tissue type. Some tissues, such as the pancreas and heart, had a lower number of genes expressed compared to the hippocampus and lungs (Figure 2B). We compared the number of genes expressed in baboon tissues with similar tissues in the Human Genotype-Tissue Expression (GTEx)[36] database downloaded from the Human Protein Atlas website. For this comparison, we used protein-coding genes expressed with levels of at least one transcript per million (TPM). The human and baboon tissues exhibit similar expression profiles across tissue types (Figure S4). The disparity in the number of genes expressed between homologous human and baboon tissues may simply be attributed to differences in library size and tissue collection. For instance, in our study, the kidney cortex was specifically sequenced, whereas the GTEx dataset refers more generally to the overall kidney. Additionally, it is important to note that the human genome, even with its greater overall autosome length, has fewer annotated genes than the baboon,[37] and the number has decreased over time.[38] Utilizing gene expression values (TPM), we defined genes as enriched in a specific tissue type if they had more than four times the average TPM compared to other tissue types. We identified a total of 2,620 genes that were enriched in at least one tissue across the 11 different tissue types studied (Table S4).

In our dataset, out of 18,644 unique protein-coding genes (Figure 2C) that were detected (TPM >0.5) (Table S5), 87% of them harbored heterozygous SNVs in transcribed regions, making these genes amenable for ASE analysis. In each tissue sample from an individual animal, an average of 60% of expressed genes contained heterozygous variants in transcribed regions. After removing heterozygous sites with less than 10 RNA-seq reads, we investigated between 3,076 and 7,660 protein-coding genes per sample (Figure 3A). Cumulatively across all 125 samples, 15,239 genes (Figure 3B) were analyzed for ASE, thus incorporating 72% of the annotated protein-coding gene set of the baboon into our ASE study (Table S6). Of the assayed genes, 1,532 unique genes (over 10%) were analyzed exclusively in a single tissue type (Figures 3C and 3D). Furthermore, over 95% of these 15,239 genes were analyzed for ASE in more than one baboon genome. The number of underlying informative variants per assayable gene varied from 1 to 97 across all 125 samples (Figure S5A). The analyzed genes had a minimum of three informative SNVs in at least one sample among the 125 samples (Figure S5B). Cumulatively, we processed 161,720 distinct SNV sites in a varying number of samples, 22,305 of which were also

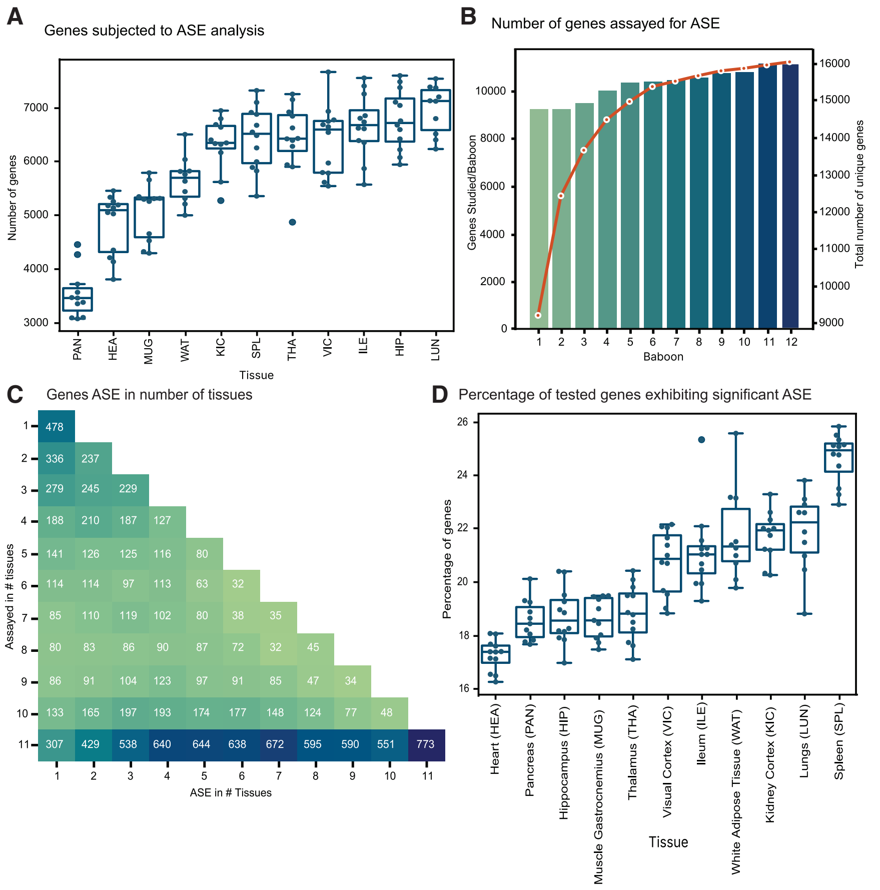

Figure 3. Extent of ASE and tissue specificity

(A) Number of genes subjected to ASE analysis across 11 different tissue types in our 12-baboon cohort. Boxes denote the interquartile range, and horizontal lines denote the median. Whiskers extend to 1.5 times the interquartile range.

(B) Number of protein-coding genes assayed for ASE in a single animal across 11 different tissue types, and the total number of genes analyzed for ASE in this study across all 12 baboons.

(C) Of the genes analyzed for ASE, 8,535 (i.e., >50% of total genes) were assayed in all 11 tissue types in any of the baboons, with the remainder assayed in a varying number of tissue types.

(D) Percentage of assayed genes as demonstrating ASE (MBASED; FDR <0.05) in a given sample, with tissues expressing a larger number of genes exhibiting a higher percentage of ASE genes. Boxes denote the interquartile range, and horizontal lines denote the median. Whiskers extend to 1.5 times the interquartile range.

See also Figures S3–S5 and Tables S6, S7, S8, S9, S10, S11, and S12.

identified in the gnomAD database version 3.1.2.[18] The majority of these SNVs were located in untranslated regions (Figure 4A) and exhibited a modifier effect (Figure 4B). Among the 24,767 missense variants we analyzed for ASE, AlphaMissense classified 706 as likely pathogenic (Figure 4C), while the PrimateAI-3D model categorized 1,709 (Figure 4D) as such.

A haplotype phased variant callset is a prerequisite to translate individual variant-level ASEs to gene-level haplotype expression. We successfully phased at least 99.7% of all identified biallelic variants into distinct haplotype blocks (Table S7). Our intention was to phase individual variants into at least gene-level haplotype blocks to study gene-level ASE events. Across all 12 baboons, we phased 96.5%–98.5% of all protein-coding genes that contained informative variants into a single haplotype block (Table S8).

Over half of the baboon annotated protein-coding genes exhibit ASE

The designation of a gene as exhibiting ASE was contingent upon the ASE attributes of the underlying informative variants found within that gene (Figure 2A). The meta-analysis-based allele-specific expression detection (MBASED)[39] method used in this study aggregates information across informative variants to identify gene-level ASE characteristics. In addition to these gene-level insights, we were interested in studying the under- or overexpression of specific SNV alleles. Therefore, we employed a β-binomial test to detect ASE at the individual SNV level. Both methods demonstrated substantial concordance in identifying strong ASE signals (see Data S1 for a detailed analysis). Essentially, MBASED exhibited greater power in detecting genes with subtle ASE signals, even when individual SNV-level signals were not statistically significant. The β-binomial method exhibited superior power in detecting ASE signals at the SNV level, even in instances where weaker gene-level ASE signals were likely attributable to heterogeneity among SNVs within a gene, potentially due to factors like alternative splicing events. For a detailed discussion on the power analysis and type I error rate of the β-binomial test, please refer to Data S1.

Across the 125 samples processed, a total of 730,673 genes were analyzed for ASE, with a median of 6,217 genes per sample. Of these 730,673 genes, 74.2% were deemed non-ASE by both tests, 13.2% were significant by both tests, 7.5% were significant only by MBASED, and the remaining 4.8% were significant only in the β-binomial test. Out of 161,720 distinct SNVs analyzed for ASE, 75,048 SNVs situated within the transcribed regions of 12,611 unique protein-coding genes were identified as exhibiting ASE in at least one sample by the β-binomial test (Table S9). Similarly, MBASED identified 13,342 unique protein-coding genes as having ASE traits (Figure 2C). Overall, 63% of the entire annotated baboon protein-coding gene set showed ASE in at least one sample (see Figure S6 for the density of ASE SNVs and genes across autosomes). Our results are in concordance with the human GTEx dataset, where 53% of protein-coding genes and 56% of genes with expression data exhibited ASE.[22] Our findings underscore the prevalent occurrence of ASE in baboon genomes. More than 89% of the genes we identified with ASE characteristics in baboons exhibited this behavior across multiple samples, with half of the ASE genes displaying ASE in at least seven samples. At the sample level,

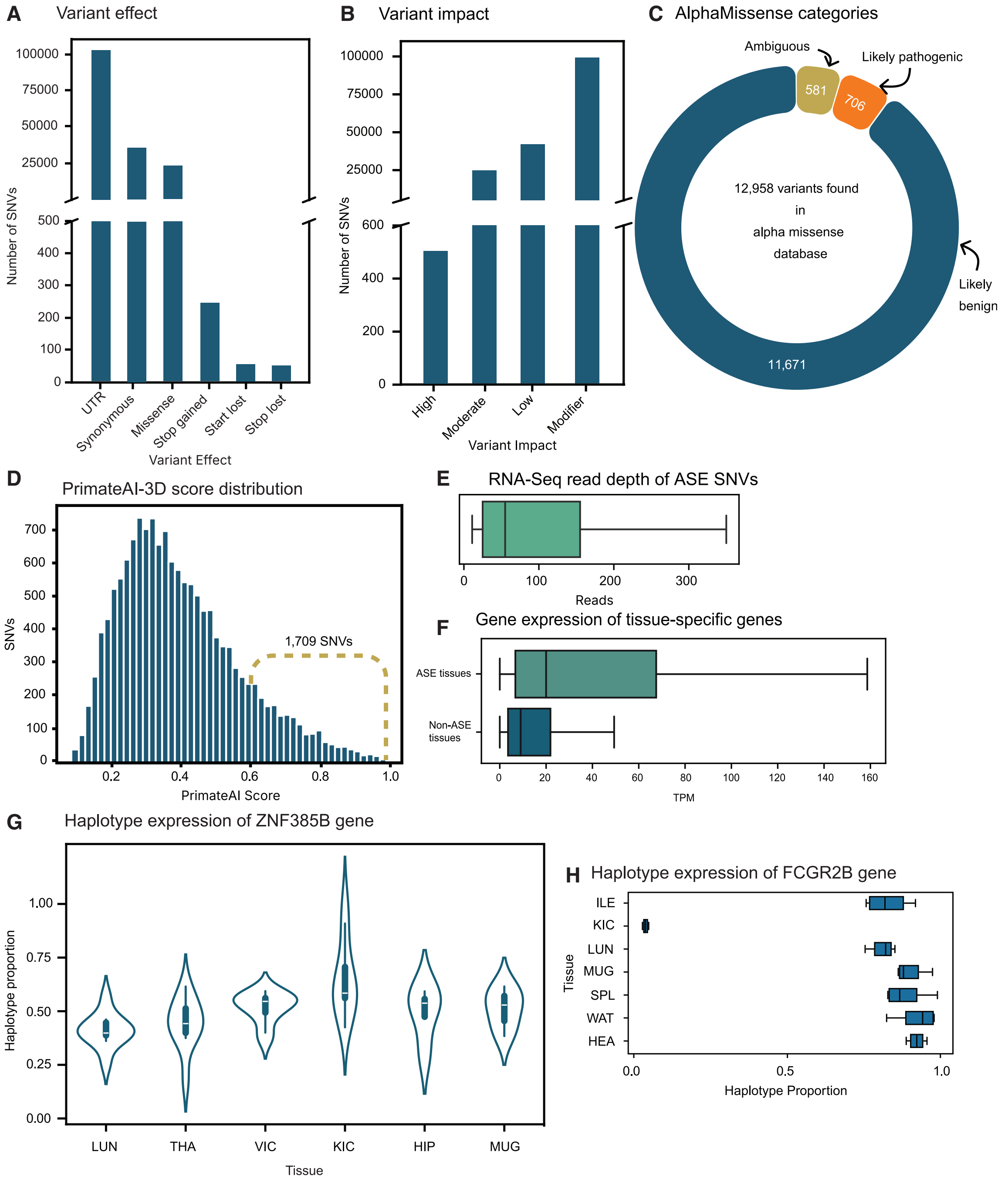

Figure 4. Impact, effect, and pathogenicity of assayed SNVs and those exhibiting ASE
(A) Effect of the analyzed SNVs in our 12-baboon cohort.
(B) Impact of the analyzed SNVs in our 12-baboon cohort.
(C) Pathogenicity of assayed variants in this study predicted by the AlphaMissense model.
(D) Pathogenicity of assayed variants in this study predicted by the PrimateAI-3D model.
(E) SNVs identified as exhibiting an ASE (β-binomial test; FDR <0.05) pattern had a minimum of 11 RNA-seq reads. Boxes denote the interquartile range, and horizontal lines denote the median. Whiskers extend to 1.5 times the interquartile range.
(F) Influence of gene expression (TPM) on the tissue specificity of ASE. Boxes denote the interquartile range, and horizontal lines denote the median. Whiskers extend to 1.5 times the interquartile range.
(G) The *ZNF385B* gene was expressed across multiple tissue types, but exhibited ASE exclusively in the kidney cortex. Boxes denote the interquartile range, and horizontal lines denote the median. Whiskers extend to 1.5 times the interquartile range.

(legend continued on next page)

MBASED identified between 573 and 1,841 protein-coding genes exhibiting ASE (Table S10). This equates to approximately 16%–26% with a median of 20% of assayable genes showing ASE (Figure 3D). SNVs exhibiting ASE traits in this study had a median of 48 reads per SNV (Figure 4E).

Our analysis revealed that with a rise in gene expression, there is an increase in the log odds (with a coefficient of 1.96) of a gene exhibiting ASE (Data S1). For genes with lower expression levels, we suspected that the β-binomial test may lack the power to conclusively reject the null hypothesis.[15,21] Our power analysis (see Data S1) also demonstrated that lower read counts (i.e., decreased expression) reduce the power of the β-binomial test to detect ASE, especially when the effect size was smaller. To accurately discern whether the observed association between gene expression levels and a pattern of ASE represents a genuine biological phenomenon or was influenced by the power limitations of the β-binomial test due to read depth, we performed a subsampling analysis. We randomly subsampled read counts from highly expressed genes at various fractions without altering the effect size, performing 100,000 iterations and rerunning the β-binomial test. In over 93.2% of iterations, the β-binomial test returned significant *p* values, suggesting that while read count has an effect, it was not the determining factor in this association. In terms of allele fold change (i.e., effect size), over 90% of genes displaying ASE characteristics showed at least a 2-fold change in read counts between reads carrying reference and alternate alleles. Conversely, more than 80% of genes with balanced expression had a fold change of less than 2.

A subset of ASE genes exhibiting tissue-specific haplotype switching

In the human genome, over half of the annotated protein-coding genes are expressed across all major tissue types.[40] However, alternative splicing and gene expression regulation often exhibit tissue specificity. *cis*-eQTLs, frequently enriched in regulatory regions, regulate this tissue specificity and often drive ASE.[8] In this study, over 93% of the assayed genes were expressed in multiple tissues, with almost half being assayed across all 11 tissue types. We hypothesized that expression levels could exhibit tissue dependence. To investigate this, we used the Kruskal-Wallis test to assess differences in read counts across tissue types for each gene. Our analysis revealed that nearly 70% of genes showed varying expression levels depending on the tissue type.

The 2,227 genes (Table S11) that exhibited ASE exclusively in a single tissue type (Figure 3C) were largely attributable to differences in gene expression levels. Although over 97% of these genes were expressed in other tissue types, their expression levels in those tissues were substantially lower (Figure 4F), resulting in decreased ASE detection power. However, we also identified genes with similar expression levels across tissues that selectively exhibited ASE in specific tissues. For example, the *ZNF385B* gene was expressed at similar levels across 56 samples, encompassing 6 different tissue types, and was also found to be expressed in multiple tissues in the Human Protein Atlas and GTEx datasets. Notably, this gene demonstrated ASE only in the kidney cortex (Figure 4G). Another key example was the *OLFM4* gene, which harbored 17 different underlying ASE variants and exhibited ASE only in the ileum across 7 different baboons. Interestingly, it preferentially expressed 1 haplotype in 5 baboons, while the other 2 baboons overexpressed the alternate haplotype. Since haplotype naming was arbitrary among individual baboons, we used SNV alleles to compare the modulation of haplotype expression across individuals. The *OLFM4* gene, known to be frequently upregulated in various types of human tumors,[41,42] was detectable in 14 different tissue types in the Human Protein Atlas dataset and 5 different tissue types in our dataset of 11 tissues. However, it exclusively exhibited ASE in ileum tissues, underscoring the remarkable tissue specificity of the ASE process.

We also identified cases of tissue-specific modulation of ASE expression within an individual baboon, presumably due to *cis*-acting variants[43] and their role in tissue-specific gene expression regulation.[8] For this analysis, we followed a similar modeling approach to a previous study,[44] including only genes that contained at least four ASE SNVs identified by the β-binomial test and exhibited ASE in at least six different tissue types within an individual baboon. We analyzed 2,510 baboon-gene pairs, representing 649 unique protein-coding genes, using a mixed β-binomial model (see star methods for details). Our aim was to determine whether a tissue-agnostic gene (Table S12), when demonstrating ASE across various tissues of the same animal, consistently preferred a specific haplotype or alternated its preference among haplotypes across different tissues. We found that 96.2% of these genes consistently favored the same haplotypes across tissue types, a pattern closely resembling that observed in humans.[44] A total of 81 protein-coding genes exhibited haplotype switching across different tissue types within individual baboons. A notable example was the *FCGR2B* gene, which expressed and exhibited ASE in ileum, kidney cortex, lungs, gastrocnemius muscle, spleen, and white adipose tissue in our dataset. Interestingly, this gene preferentially expressed one haplotype in the kidney cortex and the other haplotype in all other tissue types consistently across four different baboons (Figure 4H).

We also observed instances where genes overexpressed one haplotype in all tissue types of one animal but favored the alternate haplotype in the tissues of other animals. We were able to confidently identify 27 protein-coding genes (Figure 5A) that expressed different haplotypes in different baboons but within the same tissue type. A notable example is the *PLAGL1* gene, which harbored 14 different ASE variants and demonstrated ASE across 6 different tissue types in 8 different animals. This gene preferentially expressed one haplotype in four different animals and exhibited a preference for the alternate haplotype in the remaining four animals. The *PLAGL1* gene is known to be

(H) The *FCGR2B* gene was expressed and exhibited ASE in seven different tissues, preferentially favoring one haplotype in the kidney cortex and the other haplotype in the remaining tissues. Boxes denote the interquartile range, and horizontal lines denote the median. Whiskers extend to 1.5 times the interquartile range.
See also Figure S6 and Table S13.

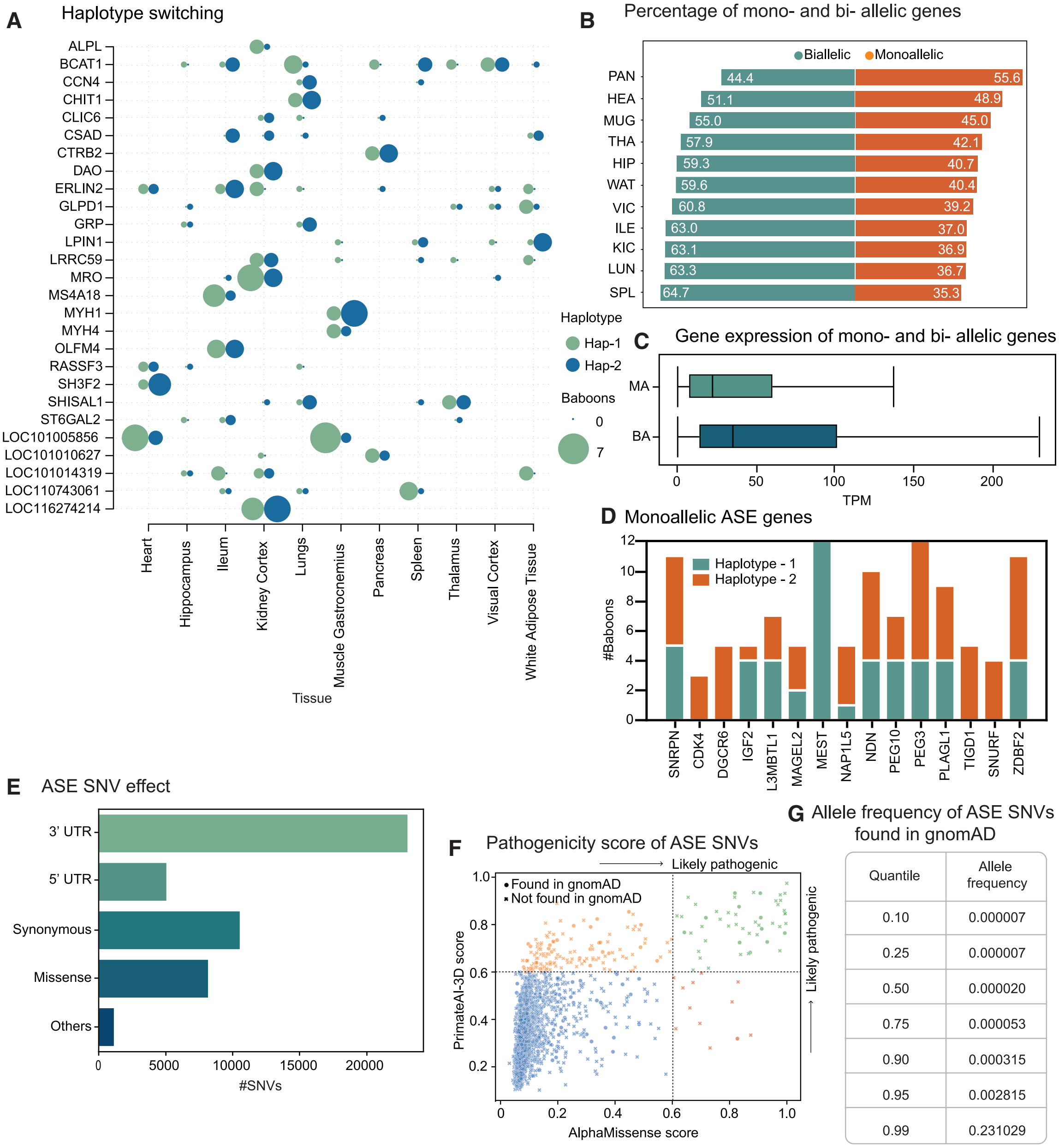

Quantile	Allele frequency
0.10	0.000007
0.25	0.000007
0.50	0.000020
0.75	0.000053
0.90	0.000315
0.95	0.002815
0.99	0.231029

Figure 5. ASE types and pathogenicity of ASE SNVs

(A) Haplotype switching between baboons with the same tissue type was observed, where haplotypes 1 and 2 are randomly determined and consistent within a gene but not between genes, with the circle size dependent on the number of baboons.

(B) Tissues exhibiting fewer expressed genes, and a reduced number of ASE genes (MBASED; FDR <0.05) predominantly contained genes that displayed monoallelic expression, attributed to the prevalence of low-expressed genes in tissues with fewer expressed genes.

(C) Monoallelic expression was common among genes with low expression levels. Boxes denote the interquartile range, and horizontal lines denote the median. Whiskers extend to 1.5 times the interquartile range.

(D) We identified 277 genes exhibiting monoallelic expression, 14 of which were previously known to show imprinting in other species. The 15 genes are shown in this plot.

(E) Most of the ASE variants (β-binomial test; FDR <0.05) found in coding regions were synonymous, followed by missense variants.

(F) Of the ASE variants (β-binomial test; FDR <0.05), 50 SNVs were categorized as likely pathogenic by both the AlphaMissense and PrimateAI-3D models.

(G) ASE variants (β-binomial test; FDR <0.05) common between baboons and humans were scarce in the gnomAD database.

See also Tables S14 and S15.

paternally imprinted in various species,[45,46] leading us to speculate that one haplotype of this gene was paternally inherited in four baboons, while a different haplotype was paternally inherited in another set of four baboons. However, the absence of specific parental haplotype information hindered any further analysis regarding gene imprinting.

Genes exhibiting monoallelic expression with known imprinting behavior

Genes exhibiting ASE traits could be either biallelic or monoallelic (see Table S13 for the list). To be classified as biallelic, a gene must generate RNA-seq reads containing both reference and alternate alleles, while being monoallelic implies the complete absence of one of the alleles in RNA-seq reads. In this study, over 68% of protein-coding genes consistently showed biallelic expression across all samples in which the gene exhibited ASE. Genes with biallelic expression tend to maintain the same pattern of expression across tissues.[43] On a sample level, genes with biallelic expression were predominant, although this varied based on the underlying tissue type (Figure 5B). Tissues with a higher number of expressed genes, such as the spleen and lungs, predominantly exhibited biallelic expression. On average, genes that displayed biallelic expression had 1.5 times more TPM than those with monoallelic expression (Figure 5C). This suggests that biallelic expression is enriched among genes with higher expression levels.[47] As our power analysis indicates, monoallelic ASE genes are more readily detectable even with fewer reads compared to biallelic ASE genes, due to the maximum effect size observed in monoallelic genes.

To identify imprinted genes, we focused on monoallelic ASE genes with at least four underlying ASE SNVs, identified using the β-binomial test and phased into a single haplotype block. We identified 277 genes exhibiting ASE that were consistently monoallelic within individual baboons across all tissue types in which the gene exhibited ASE. Notably, 85 of these ASE genes were consistently monoallelic across all samples (baboons by tissue) in which the genes exhibited ASE. Among these, we identified 14 monoallelic ASE genes (listed in Table S14) with known imprinting behavior in other species that consistently expressed the same haplotype across all samples. For instance, the *MEST* gene, expressed across 11 different tissue types in 12 baboons, consistently expressed the same haplotype across 89 resulting samples (Figure 5D). This gene has been shown to be imprinted in eight different species, including humans. We also observed genes that switched haplotypes between baboons, presumably due to *cis*-acting regulatory variants. For example, the *SNRPN* gene displayed monoallelic expression across 67 samples, encompassing 11 different tissue types and 12 different baboons. However, there was a distinct switch in haplotype preference: five baboons consistently expressed one haplotype across all tissues, while the remaining seven baboons consistently expressed the alternative haplotype.

ASE variants with biomedical significance

While the pattern of transcript abundance for individual nucleotide variants provided insight into the ASE of protein-coding genes, it is equally crucial to examine the impact of ASE variants on the functionality of these protein-coding genes. Over 60% of the ASE variants identified in this study were in untranslated regions (UTRs), and the remaining 40% of the variants were categorized as either synonymous or missense types (Figure 5E). Most ASE UTR variants were in the 3′ UTR, which is consistent with its larger size compared to the 5′ UTR. The 3′ UTR variants are often strongly associated with gene expression regulation and ASE.[48] Using the SnpEff annotation, 252 ASE variants found within 224 protein-coding genes were designated as high-impact variants. Notably, most of these high-impact variants were categorized as either stop gained, start gained, stop lost, or start lost. Over 73% of the genes containing these high-impact variants were ASE in multiple samples, suggesting these were reliable ASE calls. We analyzed 250 genetic variants predicted to introduce premature stop codons to determine their impact on gene expression. Using a β-binomial test, we found that 179 of these variants showed evidence of ASE in at least one sample. Strikingly, over 98% of these variants either exclusively or preferentially expressed the haplotype without the premature stop codon. This demonstrates that ASE, likely triggered by nonsense-mediated decay, is a powerful mechanism for suppressing the expression of potentially deleterious alleles containing premature stop codons. Even though UTRs and synonymous variants are often considered low impact, they can play crucial roles in modulating gene expression and affecting phenotypes.[49,50] We employed PrimateAI-3D[51] (obtained from Illumina, San Diego, CA) and AlphaMissense scores[52] to assess the potential pathogenicity of 8,167 missense variants exhibiting ASE in this study. More than half of these variants were found in each database, with 3,262 being shared between the two (Figure 5F). AlphaMissense identified 64 of the ASE variants as likely pathogenic, whereas PrimateAI-3D scored 198 of the variants above 0.6, suggesting their probable pathogenic nature (Table S15). Our goal was to determine whether the pathogenic alleles identified by AlphaMissense in our dataset showed a tendency toward overexpression or underexpression in our dataset. To test this, we categorized SNVs by expression class, quantified their distribution, and used a generalized linear mixed-effects model with a negative binomial distribution to assess interactions between expression class and pathogenicity, accounting for tissue and animal variability (see STAR Methods for more details). The model revealed that the number of ASE SNVs with underexpression of the annotated allele was higher in both likely benign and likely pathogenic variants. However, this effect was more pronounced in the likely pathogenic class. Specifically, the number of ASE SNVs with underexpressed annotated alleles was 4.6 times greater than those with overexpressed annotated alleles in the likely pathogenic group. In contrast, among likely benign ASE SNVs, underexpression occurred 1.7 times more frequently than overexpression.

Of the identified missense ASE variants, we located 6,241 in the gnomAD database with alleles that were the same as those found in baboons. The allelic frequency (Figure 5G) indicates that variants shared between baboon and human genomes are rare in the human population, as highlighted in previous studies.[51] This suggests that primate genomes, like those of baboons, could be valuable animal models for studying the ASE of

rare pathogenic variants, which is challenging to perform in humans due to the scarcity of these variants.

DISCUSSION

During the past decade, we have witnessed remarkable advancements in our understanding of the genetic underpinnings of human diseases. However, significant challenges persist, including the need to decipher the intricate relationships between noteworthy GWAS findings, tissue-specific gene expression, and disease susceptibility. Numerous factors, such as tissue- and age-dependent variations in gene expression, tissue-specific splicing, and other complexities, impede analyses and hinder progress. ASE represents one facet of this intricate landscape. Despite the increasing accessibility of RNA-seq data from large cohorts, inherent confounding factors obstruct a comprehensive understanding of ASE patterns in healthy human tissues and their influence on disease risk.

One of the barriers to a more complete understanding of healthy and pathologic gene expression is the dependence on studies[9,15,22] that use tissues from deceased humans with diverse medical histories. Hence, we emphasize the importance of complementing human studies with models that allow for more controlled research conditions. Baboons share 94% genome similarity with humans and offer the unique advantage of allowing tissue harvesting from age-matched healthy individuals. In addition, baboons have higher levels of coding sequence heterozygosity than do humans. This increases the number of mRNA sequences that exhibit variation, thus increasing the number of genes that can be assessed for ASE. These factors make baboons an ideal proxy for humans in biomedical studies.[26,29]

Analysis of the baboon model provides several new insights into ASE. We were able to test for ASE in 15,239 genes, 72% of the annotated baboon protein-coding gene set, and found that 63% of annotated genes showed ASE in at least 1 of the 125 tissue-specific samples assayed. The higher level of heterozygosity in baboons relative to humans makes this a more reliable estimate of the prevalence of ASE in the human genome than estimates based only on lower heterozygosity (i.e., lower powered) human datasets. In the baboons, 89% of genes that display ASE in one tissue did so in multiple samples. While 93% of the assayed genes were expressed in multiple tissues, 2,227 genes exhibited ASE in just one tissue type. Development of a comprehensive catalog of ASE across genes and tissues will advance our ability to investigate the impact of ASE on disease risk and identify causative variants.

The fraction of genes exhibiting ASE in this study is generally consistent with, but somewhat higher than, that reported for humans, which largely relied on the GTEx dataset. Unlike those studies that used binomial tests, we employed two independent methodologies to detect ASE patterns and found general concordance between them. We interpreted our results in light of empirically computed power analysis and type I error rates. We found ASE was not evenly distributed among expressed genes. Genes with elevated expression levels tend to display pronounced ASE, similar to the pattern observed in human[15] and bovine[53] genomes. ASE was more evident in tissues where the gene was expressed at greater levels compared to tissues where the same gene was expressed at lower levels. We also observed that monoallelic expression was predominant among less actively transcribed genes. For the majority of mammalian genes, the transcription in individual cells occurs in bursts and asynchronously, where one allele is expressed initially before transitioning to the other allele.[54] Monoallelic transcripts in individual cells are short-lived and less common among actively transcribed genes.[47]

Interestingly, we identified patterns of allele switching across different tissue types in genes that maintained consistent transcriptional activity levels. Aggressive allele switching has been observed during tumorigenesis,[39,55] as well as between healthy distinct tissue types.[53] This was likely driven by tissue-specific enhancers and feedback loops stemming from differences in the post-transcriptional stability of the haplotypes.[43] *cis*-eQTLs predominantly located in regulatory regions are tissue specific and regulate haplotype expression.[8] These tissue-specific genes are likely under greater selection pressure and often colocalize with disease loci.[12] For example, genes highlighted in this study such as ZNF385B, OLFM4, MEST, and PLAGL1 harbor pathogenic variants, making their expression patterns and tissue specificity of particular interest. In addition to PLAGL1, we demonstrated haplotype switching of other known imprinted genes across baboons. We also revealed relationships between tissue types in haplotype expression.

In summary, our study offers a more complete picture of ASE in healthy individuals, consisting of detailed exploration of ASE across 11 unique tissue types harvested from 12 age-matched male baboons. The use of the baboon animal model, with its heightened genome heterozygosity, enabled the analysis of over 16,000 protein-coding genes. Our findings shed new light on the complexities of mammalian ASE regulation and have important implications for understanding the expression patterns of variants crucial to human biomedical research.

Limitations of the study

Some factors can confound ASE analysis, including sequencing depth, reference allele mapping bias, and overdispersion. We generated a median of 25 million RNA-seq reads per sample (Table S2) and restricted our analyses to SNVs covered by at least 10 RNA-seq reads (Data S1, “power of beta-binomial test”), which may inadvertently exclude low-expression genes from ASE analyses. Future studies can address this limitation by sequencing tissues where the expression of the particular gene is higher or by using target enrichment during library preparation.

Reference allele mapping bias is another concern, as reads with alternate alleles with at least one mismatch may map less accurately than those carrying the reference allele. To address this, we exclusively used uniquely mapped reads and incorporated the WASP tool implemented in the STAR aligner into our processing pipeline. Overdispersion, where the observed variance in allelic ratios exceeds that expected under a simple binomial model, can also lead to spurious false positives. We accounted for overdispersion by calculating its level in our data and incorporating it into both the β-binomial test (for SNV-level analysis) and MBASED analysis (for gene-level ASE). Strong concordance was found between SNV-level and gene-level ASE results, further supporting the robustness of

CellPress
OPEN ACCESS

our findings. While these tools reduce reference allele mapping bias and overdispersion, they may also increase false negatives. Finally, the relatively small sample size ($n = 12$ baboon genomes) lacks the statistical power to investigate the causality of ASE. Future studies with larger sample sizes are needed to build robust *cis*-eQTL models to explore genotype-expression relationships.

RESOURCE AVAILABILITY

Lead contact

Further information and requests for resources and reagents should be directed to and will be fulfilled by the lead contact, Satchidananda Panda (satchin@salk.edu).

Materials availability

Peripheral tissue samples are available from the lead contact under a materials transfer agreement with Salk Institute for Biological Studies.

Data and code availability

Genomic DNA sequences and RNA-seq datasets generated in this study have been submitted to the NCBI SRA database under project PRJNA1063641. The codes used for ASE analysis are available to download from the GitHub repository (https://github.com/ramesh8v/baboon_ASE.git and https://doi.org/10.5281/zenodo.14933472). Variants and allele counts can be downloaded from https://doi.org/10.5281/zenodo.14933472.

ACKNOWLEDGMENTS

We thank Harshavardhan Doddapaneni, Marie-Claude Gingras, Donna Muzny, Richard Gibbs, and the sequence production teams of the Human Genome Sequencing Center, Baylor College of Medicine for their expert processing of the DNA samples analyzed here. This work was supported by grant no. W81XWH-18-1-0645 from the US Department of Defense, to S.P. and J.R. Research in the Panda lab is supported by funding from the National Cancer Institute (grant nos. CA258221 and CA014195) and the Wu Tsai Human Performance Alliance.

AUTHOR CONTRIBUTIONS

Conceptualization: S.P. and J.R. Funding acquisition: S.P. and J.R. Sample collection: L.S.M., G.B., H.D.L., O.D.-B., H.C., and S.P. Library preparation and sequencing: M.R., R.A.H., and H.D.L. Formal analysis: R.R., S.P., and J.R. Methodology: R.R., S.P., and J.R. Software: R.R. Visualization: R.R. Writing – original draft: R.R. Writing – review and editing: R.R., M.R., R.A.H., H.D.L., L.S.M., G.B., O.D.-B., H.C., J.R., and S.P.

DECLARATION OF INTERESTS

The authors declare no competing interests.

STAR★METHODS

Detailed methods are provided in the online version of this paper and include the following:

- KEY RESOURCES TABLE
- EXPERIMENTAL MODEL AND SUBJECT DETAILS
  - Animals
- METHOD DETAILS
  - DNA extraction
  - Illumina DNA library preparation and sequencing
  - Oxford nanopore DNA library preparation and sequencing
  - RNA extraction, library preparation, and sequencing
- QUANTIFICATION AND STATISTICAL ANALYSIS
  - Variant discovery
  - Variant annotation
  - Haplotype phasing
  - Baboon positions to human positions liftover
  - Allele-specific expression analysis
  - Gene expression quantification
  - Modeling tissue-specific haplotype switching
  - Modeling the expression of AlphaMissense annotated pathogenic alleles
  - Statistical analysis and data visualization

SUPPLEMENTAL INFORMATION

Supplemental information can be found online at https://doi.org/10.1016/j.xgen.2025.100823.

Received: August 23, 2024
Revised: December 13, 2024
Accepted: March 6, 2025
Published: April 4, 2025

REFERENCES

1. Abdellaoui, A., Yengo, L., Verweij, K.J.H., and Visscher, P.M. (2023). 15 years of GWAS discovery: Realizing the promise. Am. J. Hum. Genet. *110*, 179–194.
2. Alsheikh, A.J., Wollenhaupt, S., King, E.A., Reeb, J., Ghosh, S., Stolzenburg, L.R., Tamim, S., Lazar, J., Davis, J.W., and Jacob, H.J. (2022). The landscape of GWAS validation; systematic review identifying 309 validated non-coding variants across 130 human diseases. BMC Med. Genom. *15*, 74.
3. Villard, E., Perret, C., Gary, F., Proust, C., Dilanian, G., Hengstenberg, C., Ruppert, V., Arbustini, E., Wichter, T., Germain, M., et al. (2011). A genome-wide association study identifies two loci associated with heart failure due to dilated cardiomyopathy. Eur. Heart J. *32*, 1065–1076.
4. Chen, Z., and Schunkert, H. (2021). Genetics of coronary artery disease in the post-GWAS era. J. Intern. Med. *290*, 980–992.
5. Padmanabhan, S., and Dominiczak, A.F. (2021). Genomics of hypertension: the road to precision medicine. Nat. Rev. Cardiol. *18*, 235–250.
6. Dennison, C.A., Legge, S.E., Pardiñas, A.F., and Walters, J.T.R. (2020). Genome-wide association studies in schizophrenia: Recent advances, challenges and future perspective. Schizophr. Res. *217*, 4–12.
7. Yengo, L., Sidorenko, J., Kemper, K.E., Zheng, Z., Wood, A.R., Weedon, M.N., Frayling, T.M., Hirschhorn, J., Yang, J., and Visscher, P.M.; GIANT Consortium (2018). Meta-analysis of genome-wide association studies for height and body mass index in ∼700000 individuals of European ancestry. Hum. Mol. Genet. *27*, 3641–3649.
8. GTEx Consortium; Laboratory, Data Analysis &Coordinating Center LDACC—Analysis Working Group; Statistical Methods groups—Analysis Working Group; Enhancing GTEx eGTEx groups; NIH Common Fund; NIH/NCI; NIH/NHGRI; NIH/NIMH; NIH/NIDA; Biospecimen Collection Source Site—NDRI (2017). Genetic effects on gene expression across human tissues. Nature *550*, 204–213.
9. Glinos, D.A., Garborcauskas, G., Hoffman, P., Ehsan, N., Jiang, L., Gokden, A., Dai, X., Aguet, F., Brown, K.L., Garimella, K., et al. (2022). Transcriptome variation in human tissues revealed by long-read sequencing. Nature *608*, 353–359.
10. Harwood, M.P., Alves, I., Edgington, H., Agbessi, M., Bruat, V., Soave, D., Lamaze, F.C., Favé, M.-J., and Awadalla, P. (2022). Recombination affects allele-specific expression of deleterious variants in human populations. Sci. Adv. *8*, eabl3819.
11. Cleary, S., and Seoighe, C. (2021). Perspectives on Allele-Specific Expression. Annu. Rev. Biomed. Data Sci. *4*, 101–122.

12. Arvanitis, M., Tayeb, K., Strober, B.J., and Battle, A. (2022). Redefining tissue specificity of genetic regulation of gene expression in the presence of allelic heterogeneity. Am. J. Hum. Genet. *109*, 223–239.

13. Johansson, T., Partanen, J., and Saavalainen, P. (2022). HLA allele-specific expression: Methods, disease associations, and relevance in hematopoietic stem cell transplantation. Front. Immunol. *13*, 1007425.

14. PCAWG Transcriptome Core Group; Calabrese, C., Davidson, N.R., Demircioğlu, D., Fonseca, N.A., He, Y., Kahles, A., Lehmann, K.-V., Liu, F., Shiraishi, Y., and Soulette Cameron, M. (2020). Genomic basis for RNA alterations in cancer. Nature *578*, 129–136.

15. Kravitz, S.N., Ferris, E., Love, M.I., Thomas, A., Quinlan, A.R., and Gregg, C. (2023). Random allelic expression in the adult human body. Cell Rep. *42*, 111945.

16. van Beek, D., Verdonschot, J., Derks, K., Brunner, H., de Kok, T.M., Arts, I.C.W., Heymans, S., Kutmon, M., and Adriaens, M. (2023). Allele-specific expression analysis for complex genetic phenotypes applied to a unique dilated cardiomyopathy cohort. Sci. Rep. *13*, 564.

17. Khansefid, M., Pryce, J.E., Bolormaa, S., Chen, Y., Millen, C.A., Chamberlain, A.J., Vander Jagt, C.J., and Goddard, M.E. (2018). Comparing allele specific expression and local expression quantitative trait loci and the influence of gene expression on complex trait variation in cattle. BMC Genom. *19*, 793.

18. Taliun, D., Harris, D.N., Kessler, M.D., Carlson, J., Szpiech, Z.A., Torres, R., Taliun, S.A.G., Corvelo, A., Gogarten, S.M., Kang, H.M., et al. (2021). Sequencing of 53,831 diverse genomes from the NHLBI TOPMed Program. Nature *590*, 290–299.

19. 1000 Genomes Project Consortium; Auton, A., Brooks, L.D., Durbin, R.M., Garrison, E.P., Kang, H.M., Korbel, J.O., Marchini, J.L., McCarthy, S., McVean, G.A., and Abecasis, G.R. (2015). A global reference for human genetic variation. Nature *526*, 68–74.

20. Courbage, S., Poitou, C., Le Beyec-Le Bihan, J., Karsenty, A., Lemale, J., Pelloux, V., Lacorte, J.-M., Carel, J.-C., Lecomte, N., Storey, C., et al. (2021). Implication of Heterozygous Variants in Genes of the Leptin-Melanocortin Pathway in Severe Obesity. J. Clin. Endocrinol. Metab. *106*, 2991–3006.

21. Castel, S.E., Levy-Moonshine, A., Mohammadi, P., Banks, E., and Lappalainen, T. (2015). Tools and best practices for data processing in allelic expression analysis. Genome Biol. *16*, 195.

22. Castel, S.E., Aguet, F., Mohammadi, P., GTEx Consortium; Ardlie, K.G., and Lappalainen, T. (2020). A vast resource of allelic expression data spanning human tissues. Genome Biol. *21*, 234.

23. Rozowsky, J., Gao, J., Borsari, B., Yang, Y.T., Galeev, T., Gürsoy, G., Epstein, C.B., Xiong, K., Xu, J., Li, T., et al. (2023). The EN-TEx resource of multi-tissue personal epigenomes & variant-impact models. Cell *186*, 1493–1511.e40.

24. Zhang, J.D., Hatje, K., Sturm, G., Broger, C., Ebeling, M., Burtin, M., Terzi, F., Pomposiello, S.I., and Badi, L. (2017). Detect tissue heterogeneity in gene expression data with BioQC. BMC Genom. *18*, 277.

25. Tung, J., Zhou, X., Alberts, S.C., Stephens, M., and Gilad, Y. (2015). The genetic architecture of gene expression levels in wild baboons. Elife *4*, e04729. https://doi.org/10.7554/eLife.04729.

26. Cox, L.A., Comuzzie, A.G., Havill, L.M., Karere, G.M., Spradling, K.D., Mahaney, M.C., Nathanielsz, P.W., Nicolella, D.P., Shade, R.E., Voruganti, S., and VandeBerg, J.L. (2013). Baboons as a model to study genetics and epigenetics of human disease. ILAR J. *54*, 106–121.

27. Rogers, J., and Gibbs, R.A. (2014). Comparative primate genomics: emerging patterns of genome content and dynamics. Nat. Rev. Genet. *15*, 347–359.

28. Sørensen, E.F., Harris, R.A., Zhang, L., Raveendran, M., Kuderna, L.F.K., Walker, J.A., Storer, J.M., Kuhlwilm, M., Fontsere, C., Seshadri, L., et al. (2023). Genome-wide coancestry reveals details of ancient and recent male-driven reticulation in baboons. Science *380*, eabn8153.

29. Lin, W., Wall, J.D., Li, G., Newman, D., Yang, Y., Abney, M., VandeBerg, J.L., Olivier, M., Gilad, Y., and Cox, L.A. (2024). Genetic regulatory effects in response to a high-cholesterol, high-fat diet in baboons. Cell Genom. *4*, 100509.

30. Mure, L.S., Le, H.D., Benegiamo, G., Chang, M.W., Rios, L., Jillani, N., Ngotho, M., Kariuki, T., Dkhissi-Benyahya, O., Cooper, H.M., and Panda, S. (2018). Diurnal transcriptome atlas of a primate across major neural and peripheral tissues. Science *359*, eaao0318. https://doi.org/10.1126/science.aao0318.

31. Batra, S.S., Levy-Sakin, M., Robinson, J., Guillory, J., Durinck, S., Vilgalys, T.P., Kwok, P.-Y., Cox, L.A., Seshagiri, S., Song, Y.S., and Wall, J.D. (2020). Accurate assembly of the olive baboon (Papio anubis) genome using long-read and Hi-C data. GigaScience *9*, giaa134. https://doi.org/10.1093/gigascience/giaa134.

32. Rogers, J., Raveendran, M., Harris, R.A., Mailund, T., Leppälä, K., Athanasiadis, G., Schierup, M.H., Cheng, J., Munch, K., Walker, J.A., et al. (2019). The comparative genomics and complex population history of *Papio* baboons. Sci. Adv. *5*, eaau6947.

33. Wall, J.D., Robinson, J.A., and Cox, L.A. (2022). High-Resolution Estimates of Crossover and Noncrossover Recombination from a Captive Baboon Colony. Genom. Biol. Evol. *14*, evac040. https://doi.org/10.1093/gbe/evac040.

34. Chen, S., Francioli, L.C., Goodrich, J.K., Collins, R.L., Kanai, M., Wang, Q., Alföldi, J., Watts, N.A., Vittal, C., Gauthier, L.D., et al. (2024). A genomic mutational constraint map using variation in 76,156 human genomes. Nature *625*, 92–100.

35. Statello, L., Guo, C.-J., Chen, L.-L., and Huarte, M. (2021). Gene regulation by long non-coding RNAs and its biological functions. Nat. Rev. Mol. Cell Biol. *22*, 96–118.

36. GTEx Consortium (2013). The Genotype-Tissue Expression (GTEx) project. Nat. Genet. *45*, 580–585.

37. Nurk, S., Koren, S., Rhie, A., Rautiainen, M., Bzikadze, A.V., Mikheenko, A., Vollger, M.R., Altemose, N., Uralsky, L., Gershman, A., et al. (2022). The complete sequence of a human genome. Science *376*, 44–53.

38. Amaral, P., Carbonell-Sala, S., Vega, F.M.D.L., Faial, T., Frankish, A., Gingeras, T., Guigo, R., Harrow, J.L., Hatzigeorgiou, A.G., Johnson, R., et al. (2023). The status of the human gene catalogue. Preprint at arXiv. https://doi.org/10.1126/science.abj6987.

39. Mayba, O., Gilbert, H.N., Liu, J., Haverty, P.M., Jhunjhunwala, S., Jiang, Z., Watanabe, C., and Zhang, Z. (2014). MBASED: allele-specific expression detection in cancer tissues and cell lines. Genome Biol. *15*, 405.

40. Uhlén, M., Fagerberg, L., Hallström, B.M., Lindskog, C., Oksvold, P., Mardinoglu, A., Sivertsson, Å., Kampf, C., Sjöstedt, E., Asplund, A., et al. (2015). Proteomics. Tissue-based map of the human proteome. Science *347*, 1260419.

41. Liu, W., Li, H., Aerbajinai, W., Botos, I., and Rodgers, G.P. (2022). OLFM4-RET fusion is an oncogenic driver in small intestine adenocarcinoma. Oncogene *41*, 72–82.

42. Chen, Z., Zhang, X., Xing, Z., Lv, S., Huang, L., Liu, J., Ye, S., Li, X., Chen, M., Zuo, S., et al. (2022). OLFM4 deficiency delays the progression of colitis to colorectal cancer by abrogating PMN-MDSCs recruitment. Oncogene *41*, 3131–3150.

43. Andergassen, D., Dotter, C.P., Wenzel, D., Sigl, V., Bammer, P.C., Muckenhuber, M., Mayer, D., Kulinski, T.M., Theussl, H.-C., Penninger, J.M., et al. (2017). Mapping the mouse Allelome reveals tissue-specific regulation of allelic expression. Elife *6*, e25125. https://doi.org/10.7554/eLife.25125.

44. GTEx Consortium (2020). The GTEx Consortium atlas of genetic regulatory effects across human tissues. Science *369*, 1318–1330.

45. Ahn, J., Hwang, I.-S., Park, M.-R., Hwang, S., and Lee, K. (2021). Genomic Imprinting at the Porcine PLAGL1 Locus and the Orthologous Locus in the Human. Genes *12*, 541. https://doi.org/10.3390/genes12040541.

46. Kamiya, M., Judson, H., Okazaki, Y., Kusakabe, M., Muramatsu, M., Takada, S., Takagi, N., Arima, T., Wake, N., Kamimura, K., et al. (2000). The cell cycle control gene ZAC/PLAGL1 is imprinted–a strong candidate gene for transient neonatal diabetes. Hum. Mol. Genet. *9*, 453–460.

47. Borel, C., Ferreira, P.G., Santoni, F., Delaneau, O., Fort, A., Popadin, K.Y., Garieri, M., Falconnet, E., Ribaux, P., Guipponi, M., et al. (2015). Biased allelic expression in human primary fibroblast single cells. Am. J. Hum. Genet. *96*, 70–80.

48. Romo, L., Findlay, S.D., and Burge, C.B. (2023). Regulatory features aid interpretation of 3'UTR Variants. Am J Hum Genet *111*, 350–363. https://doi.org/10.1101/2023.08.01.551549.

49. Zeng, Z., Aptekmann, A.A., and Bromberg, Y. (2021). Decoding the effects of synonymous variants. Nucleic Acids Res. *49*, 12673–12691.

50. Soukarieh, O., Meguerditchian, C., Proust, C., Aïssi, D., Eyries, M., Goyenvalle, A., and Trégouët, D.-A. (2022). Common and Rare 5'UTR Variants Altering Upstream Open Reading Frames in Cardiovascular Genomics. Front. Cardiovasc. Med. *9*, 841032.

51. Gao, H., Hamp, T., Ede, J., Schraiber, J.G., McRae, J., Singer-Berk, M., Yang, Y., Dietrich, A.S.D., Fiziev, P.P., Kuderna, L.F.K., et al. (2023). The landscape of tolerated genetic variation in humans and primates. Science *380*, eabn8153.

52. Cheng, J., Novati, G., Pan, J., Bycroft, C., Žemgulytė, A., Applebaum, T., Pritzel, A., Wong, L.H., Zielinski, M., Sargeant, T., et al. (2023). Accurate proteome-wide missense variant effect prediction with AlphaMissense. Science *381*, eadg7492.

53. Chamberlain, A.J., Vander Jagt, C.J., Hayes, B.J., Khansefid, M., Marett, L.C., Millen, C.A., Nguyen, T.T.T., and Goddard, M.E. (2015). Extensive variation between tissues in allele specific expression in an outbred mammal. BMC Genom. *16*, 993.

54. Larsson, A.J.M., Ziegenhain, C., Hagemann-Jensen, M., Reinius, B., Jacob, T., Dalessandri, T., Hendriks, G.-J., Kasper, M., and Sandberg, R. (2021). Transcriptional bursts explain autosomal random monoallelic expression and affect allelic imbalance. PLoS Comput. Biol. *17*, e1008772.

55. Boot, A., Oosting, J., Doorn, S., Ouahoud, S., Ventayol Garcia, M., Ruano, D., Morreau, H., and van Wezel, T. (2019). Allelic Switching of DLX5, GRB10, and SVOPL during Colorectal Cancer Tumorigenesis. Int. J. Genomics *2019*, 1287671–1287710.

56. Poplin, R., Ruano-Rubio, V., DePristo, M.A., Fennell, T.J., Carneiro, M.O., Van der Auwera, G.A., Kling, D.E., Gauthier, L.D., Levy-Moonshine, A., Roazen, D., et al. (2018). Scaling accurate genetic variant discovery to tens of thousands of samples. bioRxiv, 201178. https://doi.org/10.1101/201178.

57. Martin, M. (2011). Cutadapt removes adapter sequences from high-throughput sequencing reads. EMBnet. j. *17*, 10–12.

58. Vasimuddin, M., Misra, S., Li, H., and Aluru, S. (2019). Efficient Architecture-Aware Acceleration of BWA-MEM for Multicore Systems. In 2019 IEEE International Parallel and Distributed Processing Symposium (IPDPS) (IEEE), pp. 314–324.

59. Li, H. (2018). Minimap2: pairwise alignment for nucleotide sequences. Bioinformatics *34*, 3094–3100.

60. Martin, M., Patterson, M., Garg, S., O Fischer, S., Pisanti, N., Klau, G.W., Schöenhuth, A., and Marschall, T. (2016). WhatsHap: fast and accurate read-based phasing. bioRxiv, 085050. https://doi.org/10.1101/085050.

61. Dobin, A., Davis, C.A., Schlesinger, F., Drenkow, J., Zaleski, C., Jha, S., Batut, P., Chaisson, M., and Gingeras, T.R. (2013). STAR: ultrafast universal RNA-seq aligner. Bioinformatics *29*, 15–21.

62. Cingolani, P., Platts, A., Wang, L.L., Coon, M., Nguyen, T., Wang, L., Land, S.J., Lu, X., and Ruden, D.M. (2012). A program for annotating and predicting the effects of single nucleotide polymorphisms, SnpEff: SNPs in the genome of Drosophila melanogaster strain w1118. Fly *6*, 80–92 iso-2; iso-3.

63. van de Geijn, B., McVicker, G., Gilad, Y., and Pritchard, J.K. (2015). WASP: allele-specific software for robust molecular quantitative trait locus discovery. Nat. Methods *12*, 1061–1063.

64. Li, B., and Dewey, C.N. (2011). RSEM: accurate transcript quantification from RNA-Seq data with or without a reference genome. BMC Bioinf. *12*, 323.

65. Yin, L., Zhang, H., Tang, Z., Xu, J., Yin, D., Zhang, Z., Yuan, X., Zhu, M., Zhao, S., Li, X., and Liu, X. (2021). rMVP: A Memory-efficient, Visualization-enhanced, and Parallel-accelerated Tool for Genome-wide Association Study. Genom. Proteom. Bioinform. *19*, 619–628.

STAR★METHODS

KEY RESOURCES TABLE

REAGENT or RESOURCE	SOURCE	IDENTIFIER
Biological samples		
12 different tissue samples collected from 12 age matched baboons; See Table S1	Mure et al.[30]	N/A
Critical commercial assays		
Puregene DNA extraction kit	QIAGEN	Cat# 158063
PicoGreen dsDNA Quantitation Reagent	Thermo Fisher Scientific	Cat# P7589
AMPure XP beads	Beckman Coulter	Cat# A63882
KAPA Hyper kits	Roche	Cat# KK8505
TruSeq PE Cluster Generation kit	Illumina	Cat# PE-401-3001
HiSeq X^{TM} Ten Reagent Kit v2.5 (300 cycles)<br>- HiseqX V2.5 Cbot<br>- HiseqX V2.5 Flowcells<br>- HiseqX SBS Box 1 Kit<br>- HiseqX SBS Box 2 Kit<br>- HiseqX PE Kit	Illumina	Cat# FC-501-2501
Ligation Sequencing Kit	Oxford Nanopore	Cat# SQK-LSK109
RNAeasy mini kit	QIAGEN	
Trizol	Thermo Fisher Scientific	Cat# 15596026
TruSeq Stranded mRNA kit	Thermo Fisher Scientific	Cat# #20020595
QUBIT dsDNA HS Assay Kit	Thermo Fisher Scientific	Cat# Q32851
Deposited data		
Short read DNA-Seq data	This paper	BioProject: PRJNA1063641 (https://dataview.ncbi.nlm.nih.gov/object/PRJNA1063641)
Long read DNA-Seq data	This paper	BioProject: PRJNA1063641 (https://dataview.ncbi.nlm.nih.gov/object/PRJNA1063641)
Short read RNA-Seq data	This paper	BioProject: PRJNA1063641 (https://dataview.ncbi.nlm.nih.gov/object/PRJNA1063641)
VCF file containing phased variants	This paper	https://doi.org/10.5281/zenodo.14933472
Allele counts	This paper	https://doi.org/10.5281/zenodo.14933472
Software and algorithms		
GTAK v4.3.0.0	Poplin [56]	https://github.com/broadinstitute/gatk
SAMtools v1.19		https://github.com/samtools/samtools
Cutadapt v4.0	Martin [57]	https://github.com/marcelm/cutadapt
BWA-MEM2 v2.2.1	Vasimuddin et al.[58]	https://github.com/bwa-mem2/bwa-mem2
SnpEff		https://github.com/pcingola/SnpEff
SnpSift		https://github.com/pcingola/SnpSift
Minimap v2.24	Li [59]	https://github.com/lh3/minimap2
WhatsHap	Martin et al.[60]	https://github.com/whatshap/whatshap
phASE-Extender		https://github.com/everestial/phase-extender
UCSC liftover tool		https://genome.ucsc.edu/cgi-bin/hgLiftOver
STAR v2.7.1a	Dobin et al.[61]	https://github.com/alexdobin/STAR
GATK ASEReadCounter		https://gatk.broadinstitute.org/hc/en-us/articles/360037428291-ASEReadCounter
MBASED v1.38.0	Mayba et al.[39]	https://bioconductor.org/packages/release/bioc/html/MBASED.html
Scipy v1.10.0		https://github.com/scipy/scipy

(Continued on next page)

Continued

REAGENT or RESOURCE	SOURCE	IDENTIFIER
Numpy v1.25.0		https://github.com/numpy/numpy
Pandas v2.0.2		https://github.com/pandas-dev/pandas
Polars v0.18.0		https://github.com/pola-rs/polars
Statsmodels v0.14.0		https://github.com/statsmodels/statsmodels
Matplotlib v3.7.1		https://github.com/matplotlib/matplotlib
Seaborn v0.12.2		https://github.com/mwaskom/seaborn
Other		
PrimateAI-3D scores	Gao et al.[51]	Directly obtained from Illumina, San Diego
AlphaMissense scores	Cheng et al.[52]	https://console.cloud.google.com/storage/browser/dm_alphamissense;tab=objects?pli=1&prefix=&forceOnObjectsSortingFiltering=false

EXPERIMENTAL MODEL AND SUBJECT DETAILS

Animals

For this study, we repurposed baboon tissue samples initially collected in our previous research, detailed in.[30] In brief, 12 male olive baboons (*Papio anubis*), aged 5 to 6 years and weighing 7 to 11 kg, were acclimated to a 12-h light-dark cycle for one month before euthanasia. The animals had continuous access to water and were fed fruits and primate meal pellets twice daily. Perfusion was conducted via the left ventricle using 4 L of ice-cold (2°C) buffered, oxygenated Ame's solution. The tissue samples were immediately frozen in dry ice and stored at −80°C, ensuring the entire process from collection to storage was completed within 120 min.

METHOD DETAILS

DNA extraction

DNA was extracted from baboon prefrontal cortex using Puregene DNA extraction kit. We used two independent methods for estimating the quantity and quality of the DNA before library construction: i) PicoGreen dsDNA Quantitation Reagent used for DNA quantification and ii) Semi-quantitative and qualitative gels were used to estimate DNA sample integrity. These gels indirectly provide a "cross-validation" for the PicoGreen assay. The appropriate QC intake information was imported into our LIMS system including sample location, sample 2D tube barcode, genome center sample name, sample provider's sample designation, species, sample type and relevant sample HGSC QC data (concentration and volume). Other metadata including sex and other information were also recorded.

Illumina DNA library preparation and sequencing

Genomic DNA samples that passed quality control were used to produce Illumina paired-end libraries following the manufacturer's protocol with the modifications described here. These libraries were prepared on Beckman robotic workstations (Biomek FX and FXp models) (Beckman Coulter Inc. Brea CA). Briefly, genomic DNA (0.75 μg in 70 μL volume) was sheared to roughly 350–400 base pair fragments using Covaris plates and the E210 system (Covaris, Inc. Woburn, MA). This was followed by purification of sheared DNA using the AMPure XP beads, double SPRI bead size selection with different ratios of AMPure XP beads (0.57X/0.75X), generating for each library a narrow band of DNA fragment size. Employing KAPA Hyper kits (KK8505), DNA samples were end-repaired and underwent 3′-adenylation in the same reaction. We then ligated barcoded adaptors, resulting in PCR-Free libraries. An array of 8-bp barcoded adapters (n = 96, Dual Index Barcode from IDT) were used for sample barcoding. After the ligation step, the libraries were twice cleaned using 0.8X AMPure XP beads to remove leftover adapters/adapter dimers. The final size of library inserts was estimated and quantified on a Fragment Analyzer (Agilent/Advance Analytical, Inc) system using electrophoresis methods and an Applied Biosystems 7900HT Fast Real-Time PCR System to achieve an average final library size of approximately 530bp. Minimum concentration is set as >1.8nM and minimum library size >470 bp.

We used Illumina's cBot cluster generation system with TruSeq PE Cluster Generation Kits (Cat. no. PE-401-3001) to prepare DNA libraries for sequencing. The libraries are first denatured, then loaded into a HiSeq flow cell (one lane) following manufacturer's recommendations. Each lane is routinely spiked with a control library of 1% phiX to allow evaluation of sequencing quality. Runs on the sequencing instrument were performed using paired-end mode on the Illumina HiSeqX platform and TruSeq SBS Kits (Cat. no. FC-401-3001). This produces data through the sequencing-by-synthesis approach. Between 300 and 400 million successful reads are generated in a single flow cell lane, producing 37 to 40 Gb per library pool.

Oxford nanopore DNA library preparation and sequencing

The appropriate libraries for Oxford nanopore long-read sequencing were generated using the Ligation Sequencing Kit from ONT (SQK-LSK109) and sequenced using R9 flow cells (FLO-PRO002, ONT). This was done using PromethION devices from Oxford Nanopore. Default parameters were employed in analysis. The libraries contained 500ng of subject DNA with 16kb average insert size. The libraries were loaded into the ONT flow cells, and the sequence data were obtained over a period of 72 h.

RNA extraction, library preparation, and sequencing

Total RNA was extracted from each tissue sample using methods optimized for the specific tissue type. The RNeasy Mini kit from QIAGEN was employed for most tissues, following the manufacturer's protocol for RNA isolation and purification. For samples with high-fat content, Trizol (ThermoFisher Scientific) was utilized. For a detailed description of RNA isolation, please refer to.[30] Library preparation was conducted using the TruSeq Stranded mRNA kit (Illumina, San Diego), adhering to the provided guidelines. We started with 500 ng of total RNA, which underwent poly-A selection using beads, fragmentation through metal-ion hydrolysis, and conversion into double-stranded (ds) cDNA. This cDNA was then processed for end-repair, A-tailing, adapter ligation using either TruSeq CD indexes (Illumina, San Diego) or IDT for Illumina-TruSeq UD indexes (Illumina, San Diego), followed by 15 cycles of PCR amplification. Post-preparation, libraries were quantified with the QUBIT dsDNA HS Assay Kit (ThermoFisher Scientific). After pooling, the libraries were sequenced on Illumina NovaSeq 6000 platform at the NGS Core Facility of the Salk Institute.

QUANTIFICATION AND STATISTICAL ANALYSIS

Variant discovery

We adhered to the GATK germline variant calling[56] best practices (https://gatk.broadinstitute.org/hc/en-us/articles/360035535932-Germline-short-variant-discovery-SNPs-Indels-) to identify SNVs and indels in 12 wild baboon genomes. In summary, we trimmed Illumina PE150 reads of adapters with Cutadapt v4.0[57] and aligned them to the *Papio anubis* v1.0 reference genome[31] using the BWA-MEM2 v2.2.1 aligner.[58] Duplicate reads in the BAM file were marked using GATK MarkDuplicatesSpark. For each sample, variants were identified using GATK HaplotypeCaller in GVCF format. Subsequently, GenotypeGVCF was employed to jointly call variants for all 12 samples. We applied the GATK hard filter (SNPs: "QD $<$ 2.0 || FS $>$ 60.0 || MQ $<$ 40.0 || MQRankSum < -12.5 || ReadPosRankSum < -8.0"; Indels: "QD $<$ 2.0 || FS $>$ 200.0 || ReadPosRankSum < -20.0") to filter variants, discarding any that didn't meet the criteria. After recalibrating base quality scores with GATK BaseRecalibrator and applying them to the BAM files, we called and filtered variants once more using the aforementioned filters.

Variant annotation

We employed SnpEff[62] to annotate the high-quality SNVs that were filtered in the preceding step. To establish the SnpEff database, we utilized the *Papio anubis* v1.0 reference genome along with annotations in GTF format. While running the Eff command, we maintained default settings but included the "-no-downstream -no-intergenic -no-upstream" flags to exclude downstream, intergenic, and upstream annotations. For downstream processing, we used SnpSift extractFields to convert fields from the annotated VCF file into a tab-delimited format.

Haplotype phasing

ONT reads from 12 samples were aligned to the *P. anubis* v1.0 genome[31] using minimap v2.24.[59] For each sample, WhatsHap[60] was run with default settings, taking three inputs: 1) the bam file resulting from ONT reads alignment; 2) the bam file from Illumina PE150 reads; and 3) the VCF file generated during the variant calling process. WhatsHap employs read-based phasing to segregate variants into haplotype blocks. The phased VCF file from WhatsHap for each sample was then processed through phASE-Extender (https://github.com/everestial/phase-extender) for five consecutive iterations using default parameters, aiming to further extend the haplotype blocks for each sample. Our aim was to compare haplotype expression across multiple animals. Therefore, we assigned 'Haplotype-1′ and 'Haplotype-2′ arbitrarily based on shared alleles, as most genes have multiple SNVs shared among animals. These arbitrary names are not comparable between different genes, but they are consistent for comparison across animals and tissues within the same gene. This approach allowed us to investigate haplotype-specific expression preferences across animals.

Baboon positions to human positions liftover

SNV positions from the baboon assembly (GCF_008728515.1) were converted to the hg38 human assembly coordinates using the UCSC liftover tool. The necessary chain files for the conversion were downloaded from https://hgdownload.soe.ucsc.edu/hubs/GCF/008/728/515/GCF_008728515.1/liftOver/GCF_008728515.1ToHg38.over.chain.gz (accessed on 07/31/2023) and https://hgdownload.soe.ucsc.edu/goldenPath/hg38/liftOver/hg38ToGCF_008728515.1.over.chain.gz (accessed on 11/27/2023). PrimateAI-3D scores used in this study were directly obtained from Illumina Inc., San Diego. AlphaMissense scores were downloaded from the Google cloud bucket (https://console.cloud.google.com/storage/browser/dm_alphamissense;tab=objects?pli=1&prefix=&forceOnObjectsSortingFiltering=false; accessed on 07/31/2023).

Allele-specific expression analysis

RNA-Seq Illumina PE150 reads underwent adapter trimming with Cutadapt v4.0, and any reads shorter than 50 bases were discarded. These trimmed reads from 125 samples were individually aligned to the *P. anubis* v1.0 reference genome using the STAR v2.7.1a aligner.[61] While STAR was used with its default settings, the –waspOutputMode flag was added to enable WASP mode.[63] Only reads that passed the WASP filter, specifically those marked with "vW:i:1", were retained in the BAM file, while the rest were excluded. This curated BAM file was then utilized in GATK ASEReadCounter[21] to count the RNA-Seq reads that carried either reference or alternate alleles at heterozygous biallelic locations for each sample. Duplicated reads and reads with secondary alignments were not considered for ASE counting.

In our ASE analysis, we aimed to identify high-quality ASE SNVs and genes while minimizing false positives. Heterozygous locations with fewer than ten RNA-Seq reads were considered unreliable for ASE analysis and were therefore excluded from further evaluation. To assess reference bias, we calculated the ratio of reads containing the reference allele to the total number of reads mapped to each SNV. This analysis allowed us to understand the extent of reference bias within our data and establish an empirical null hypothesis. A median of 0.5 was recorded as reference bias, reflecting a negligible bias, if any exists. We then computed the dispersion in our dataset. To do this, we selected only SNVs with at least ten reads that expressed both alleles in at least one sample among the 125 samples assayed. The median dispersion was 0.00098, and over 80% of SNVs had less than 0.0018. Hence, we used 0.001 as a constant dispersion in the ASE analysis.

We used MBASED R package followed by a Benjamini-Hochberg multiple testing correction (FDR <0.05) to determine the allelic imbalance at each protein-coding gene. MBASED aggregates haplotype-phased SNV-level information into gene-level using meta-analysis and provides the estimate of ASE and a corresponding *p*-value. In addition to gene-level ASE information, we used a two-tailed beta-binomial test, followed by a Benjamini-Hochberg multiple testing correction (FDR <0.05) to get SNV-level ASE information. Heterozygous SNV sites with an FDR less than 0.05 were identified as having statistically significant SNV-level allele-specific expression. We processed each sample (i.e., each animal-tissue combination) independently for both gene-level MBASED analysis and SNV-level beta-binomial testing. Consequently, FDR correction was applied by aggregating the *p*-values from all genes assayed by MBASED within each sample, and separately aggregating the *p*-values for all heterozygous SNV sites from the beta-binomial tests.

To assess the reliability of our statistical approach, we conducted a power analysis to determine the probability of the beta-binomial test correctly rejecting the null hypothesis when a true effect was present. We simulated datasets with varying allelic proportions (effect sizes from 0.01 to 0.99) and read depths (200, 100, 75, 50, 25, and 10 reads), representing the total number of reads aligned to a given locus. The simulations were performed using a dispersion parameter (ρ) of 0.001, which we estimated previously. For each combination of effect size and read depth, we simulated 10,000 datasets under the alternate hypothesis of allelic imbalance. We then calculated power as the proportion of these datasets where the beta-binomial test correctly rejected the null hypothesis at a significance level of 0.05. We computed *p*-values using the cumulative distribution function (CDF) of the beta-binomial distribution. We considered the test to be two-tailed, calculating the *p*-value as twice the minimum of the CDF value and its complement. This analysis enabled us to determine the probability of detecting true allelic imbalance as a function of both the magnitude of the imbalance and the sequencing depth.

Gene expression quantification

To compare baboon gene expression with that of humans, we used probability model-based quantifiers for transcriptomic mapping to quantify baboon gene expression. First, RNA-Seq Illumina PE150 reads were adapter-trimmed using Cutadapt v4.0, and any reads shorter than 50 bases were discarded. Next, we ran RSEM[64] with default settings to map RNA-Seq reads onto the baboon reference genome and quantify expression in TPM values. We then compared the number of genes expressed in baboon tissues with those expressed in similar tissues from the Human GTEx 36 database, downloaded from the Human Protein Atlas website (https://www.proteinatlas.org/about/download; accessed on 07/31/2023). We followed EBI expression atlas criteria (https://www.ebi.ac.uk/gxa/FAQ.html; accessed on 07/31/2023) to categorize gene expression levels as Low (between 0.5 and 10 TPM), Medium (between 10 and 1000 TPM), and High (more than 1000 TPM).

Modeling tissue-specific haplotype switching

We used a beta-binomial mixture model to evaluate tissue-specific haplotype switching within individual baboons. Our modeling approach closely mirrored that of a previously published study. For this analysis, we focused exclusively on ASE genes that met two criteria: they had at least four underlying ASE SNVs identified through a beta-binomial test and exhibited ASE in a minimum of six distinct tissue types within a single baboon. We separately aggregated the read counts for haplotype-1 and haplotype-2 alleles and incorporated them into the beta-binomial mixture model.

The mixture model assumes K components, each characterized by an allelic ratio α_k and a shared overdispersion parameter ϕ. Let x_i represent the aggregated number of reads associated with one haplotype and y_i the total number of reads in tissue i. The likelihood for the model is expressed as:

$$L(x; y|\pi, \alpha, \phi) = \prod_{i=1}^{N} \sum_{k=1}^{K} \pi_k \mathrm{BB}(x_i; y_i; \alpha_k, \phi),$$

Where π_k is the weight for the $k - th$ component $(\sum_{k=1}^{K} \pi_k = 1)$, and BB represents the beta-binomial probability mass function parameterized by α_k and ϕ.

Parameter estimation was performed using constrained optimization to maximize the log likelihood. For $K = 1$, the optimization involved estimating α_1 and ϕ . For > 1 , additional parameters included α_k for each component and the mixture weights π_k. The optimization employed multiple initial parameter values to ensure robustness, with the best solution selected based on maximum likelihood. Constraints were applied to ensure $\pi_k > 0$, $\sum_{k=1}^{K} \pi_k = 1$, and $0 < \alpha_k < 1$

To determine the optimal number of components (K), we utilized a model selection approach based on likelihood ratio tests (LRT). Starting with $K = 1$, we incrementally increased K, testing whether the more complex model provided a significantly better fit. We compared nested models using a chi-squared test, evaluating the log likelihood ratio at a significance level of $p < 0.05$. The resulting model provided estimates for the allelic ratios (α_k) the overdispersion parameter (ϕ) , and the relative contributions of each mode (π_k).

Modeling the expression of AlphaMissense annotated pathogenic alleles

We aimed to assess whether pathogenic alleles, as categorized by AlphaMissense in our dataset, were more likely to be overexpressed or underexpressed. To achieve this, we first classified each assayed biallelic SNV into one of two expression categories—underexpressed or overexpressed—based on the expression of the annotated allele relative to the other allele. Next, we quantified the number of SNVs in each expression category for each sample. Using a binomial test, we established the null hypothesis that the frequency of underexpressed SNVs is not significantly different from that of overexpressed SNVs in a sample.

Recognizing the count-based nature of the data and its overdispersion, we applied a generalized linear mixed-effects model (GLMM) using a negative binomial distribution. The model incorporated fixed effects for expression class (overexpressed or underexpressed), Alphamissense pathogenic classification of ASE SNVs (likely benign or likely pathogenic), and their interaction to examine whether the effect of expression class varied by variant classification. To account for variability across tissues and animals, random intercepts were included for both tissue type and baboon. We fitted the model using the glmer.nb() function from the lme4 package in R, and post-hoc analyses were performed using the emmeans package.

Statistical analysis and data visualization

Data analyses, both statistical and downstream, were conducted in the Python data analysis environment primarily using the following packages: Scipy, Numpy, Pandas, Polars, Statsmodels, Matplotlib, and Seaborn. Variant and gene density ideograms were created using the CMplot R package.[65]

Cell Genomics, Volume *5*

Supplemental information

Genome-wide allele-specific expression

in multi-tissue samples from healthy male baboons

reveals the transcriptional complexity of mammals

Ramesh Ramasamy, Muthuswamy Raveendran, R. Alan Harris, Hiep D. Le, Ludovic S. Mure, Giorgia Benegiamo, Ouria Dkhissi-Benyahya, Howard Cooper, Jeffrey Rogers, and Satchidananda Panda

1. Empirical null hypothesis

The assumed null hypothesis for our analysis was an allelic ratio of 0.5, anticipating that half of the mapped reads would carry the reference allele and the other half the alternate allele. Any statistically significant deviation from this ratio was considered indicative of allele-specific expression (ASE). However, various technical confounders, including read mapping bias, can skew this assumption. Reads carrying the alternate allele inherently possess at least one more mismatch compared to those carrying the reference allele, potentially reducing their chances of mapping correctly. To mitigate this potential bias, we enabled the WASP mode in STAR mapping using the "–waspOutputMode" flag. Subsequently, we retained only those reads specifically marked with the "vW:i:1" flag. We then computed an empirical null hypothesis by comparing the number of reads carrying the reference allele versus the alternate allele across all heterozygous loci within each sample.

For our analysis, we focused exclusively on heterozygous SNVs located within the transcribed regions of protein-coding genes, requiring a minimum of ten RNA-Seq reads mapped to each SNV. To evaluate potential mapping bias, we calculated the reference bias for each individual SNV by dividing the number of reads carrying the reference allele by the total number of reads mapped. Across all 125 samples and all SNVs examined, the median reference bias was 0.5 (**Figure SD1**), suggesting that mapping bias, if present, was effectively controlled. At the individual sample level, the median reference bias remained at 0.5 in all except pancreatic samples (see the table below).

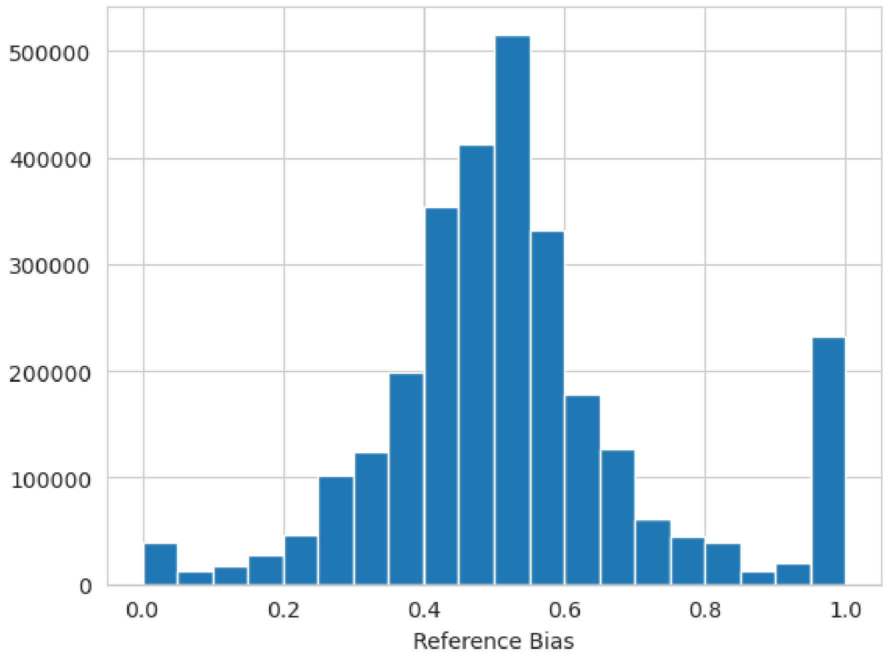

Figure SD1: Histogram illustrating the distribution of reference bias across all SNVs and samples.

Animal	Tissue	Median Reference Bias
Baboon-1	HEA	0.5000
Baboon-1	HIP	0.5000
Baboon-1	ILE	0.5000
Baboon-1	LUN	0.5000
Baboon-1	MUG	0.5000
Baboon-1	PAN	0.5217
Baboon-1	SPL	0.5000
Baboon-1	THA	0.5000
Baboon-1	VIC	0.5000
Baboon-1	WAT	0.5021
Baboon-2	HEA	0.5034
Baboon-2	HIP	0.5000
Baboon-2	ILE	0.5000
Baboon-2	KIC	0.5000
Baboon-2	LUN	0.5000
Baboon-2	MUG	0.5067
Baboon-2	PAN	0.5172
Baboon-2	SPL	0.5000
Baboon-2	THA	0.5000
Baboon-2	VIC	0.5000
Baboon-2	WAT	0.5049
Baboon-3	HEA	0.5052
Baboon-3	HIP	0.5000
Baboon-3	ILE	0.5000
Baboon-3	KIC	0.5000
Baboon-3	LUN	0.5000
Baboon-3	MUG	0.5000
Baboon-3	PAN	0.5041
Baboon-3	SPL	0.5050
Baboon-3	THA	0.5000
Baboon-3	VIC	0.5000
Baboon-4	HEA	0.5000
Baboon-4	HIP	0.5000
Baboon-4	ILE	0.5000
Baboon-4	KIC	0.5000
Baboon-4	MUG	0.5027
Baboon-4	PAN	0.5143
Baboon-4	SPL	0.5055

Baboon-4	THA	0.5000
Baboon-4	VIC	0.5000
Baboon-5	HEA	0.5000
Baboon-5	HIP	0.5000
Baboon-5	ILE	0.5000
Baboon-5	KIC	0.5000
Baboon-5	LUN	0.5000
Baboon-5	MUG	0.5091
Baboon-5	PAN	0.5097
Baboon-5	SPL	0.5056
Baboon-5	THA	0.5000
Baboon-5	VIC	0.5000
Baboon-5	WAT	0.5049
Baboon-6	HEA	0.5000
Baboon-6	HIP	0.5000
Baboon-6	ILE	0.5000
Baboon-6	KIC	0.5000
Baboon-6	LUN	0.5000
Baboon-6	SPL	0.5000
Baboon-6	THA	0.5000
Baboon-6	VIC	0.5000
Baboon-6	WAT	0.5000
Baboon-7	HEA	0.5000
Baboon-7	HIP	0.5000
Baboon-7	ILE	0.5000
Baboon-7	KIC	0.5000
Baboon-7	LUN	0.5000
Baboon-7	MUG	0.5030
Baboon-7	PAN	0.5082
Baboon-7	SPL	0.5000
Baboon-7	THA	0.5000
Baboon-7	VIC	0.5000
Baboon-7	WAT	0.5000
Baboon-8	HEA	0.5000
Baboon-8	HIP	0.5000
Baboon-8	ILE	0.5021
Baboon-8	KIC	0.5000
Baboon-8	LUN	0.5000
Baboon-8	MUG	0.5000
Baboon-8	PAN	0.5227
Baboon-8	SPL	0.5000

Baboon-8	THA	0.5000
Baboon-8	VIC	0.5000
Baboon-8	WAT	0.5033
Baboon-9	HEA	0.5000
Baboon-9	HIP	0.5000
Baboon-9	ILE	0.5000
Baboon-9	KIC	0.5000
Baboon-9	LUN	0.5000
Baboon-9	MUG	0.5000
Baboon-9	PAN	0.5000
Baboon-9	SPL	0.5000
Baboon-9	THA	0.5000
Baboon-9	VIC	0.5000
Baboon-9	WAT	0.5000
Baboon-10	HEA	0.5000
Baboon-10	HIP	0.5000
Baboon-10	ILE	0.5000
Baboon-10	KIC	0.5000
Baboon-10	MUG	0.5000
Baboon-10	PAN	0.5039
Baboon-10	SPL	0.5016
Baboon-10	THA	0.5000
Baboon-10	VIC	0.5000
Baboon-10	WAT	0.5000
Baboon-11	HEA	0.5000
Baboon-11	HIP	0.5000
Baboon-11	ILE	0.5000
Baboon-11	KIC	0.5000
Baboon-11	LUN	0.5000
Baboon-11	MUG	0.5000
Baboon-11	PAN	0.5152
Baboon-11	SPL	0.5000
Baboon-11	THA	0.5000
Baboon-11	VIC	0.5000
Baboon-11	WAT	0.5085
Baboon-12	HEA	0.5000
Baboon-12	HIP	0.5000
Baboon-12	ILE	0.5000
Baboon-12	KIC	0.5000
Baboon-12	LUN	0.5000
Baboon-12	MUG	0.5000

Baboon-12	PAN	0.5060
Baboon-12	SPL	0.5000
Baboon-12	THA	0.5000
Baboon-12	VIC	0.5000
Baboon-12	WAT	0.5006

Table SD1: Median reference bias across all assayed SNVs in a sample.

2. Empirical dispersion estimation

Overdispersion significantly impacts ASE estimation, affecting both independent beta-binomial tests and MBASED analysis. To quantify the dispersion in our data, we selected heterozygous SNVs within transcribed regions of protein-coding genes, each supported by at least ten RNA-Seq reads. To ensure robust dispersion estimation, we focused on biallelic SNVs expressed in at least five samples. This criterion ensured that the selected SNVs expressed both reference and alternate alleles in at least one of the 125 analyzed samples. We employed the log-likelihood method of the beta-binomial distribution, implemented in the Python SciPy package, to calculate the dispersion within our dataset. Based on the resulting dispersion distribution, we applied a uniform dispersion value of 0.001 for both MBASED and the beta-binomial test.

Decile	0.1	0.2	0.3	0.4	0.5	0.6	0.7	0.8	0.9
Dispersion	0.000079	0.00026	0.00045	0.00062	0.00078	0.00093	0.0011	0.0013	0.0019

Table SD2: Distribution of empirically estimated dispersion.

3. Power of beta-binomial test

We hypothesized that the power of the beta-binomial test to identify ASE would be largely influenced by both the effect size and the number of mapped reads. To investigate this relationship, we simulated datasets with varying allelic proportions, representing effect sizes from 0.01 to 0.99, and read depths of 200, 100, 75, 50, 25, and 10 reads. These read depths represent the total number of reads aligned to a given locus. Our simulations were designed to closely reflect the empirical read depths observed in our data (**Table SD3**), as well as the range of effect sizes identified (**Figure SD1**). The simulations were conducted using a dispersion parameter (ρ) of 0.001. We generated 10,000 datasets for each combination of effect size and read depth, simulating data under the alternative hypothesis of allelic imbalance. Power was

then calculated as the proportion of these datasets where the beta-binomial test successfully rejected the null hypothesis at a significance level of 0.05. We computed p-values using the cumulative distribution function (CDF) of the beta-binomial distribution. Given the two-tailed nature of the test, the p-value was determined as twice the minimum value between the CDF value and its complement.

Decile	0.1	0.2	0.3	0.4	0.5	0.6	0.7	0.8	0.9
#reads mapped	12	16	20	26	34	46	64	96	175

Table SD3: Distribution of number of reads mapped to a given locus.

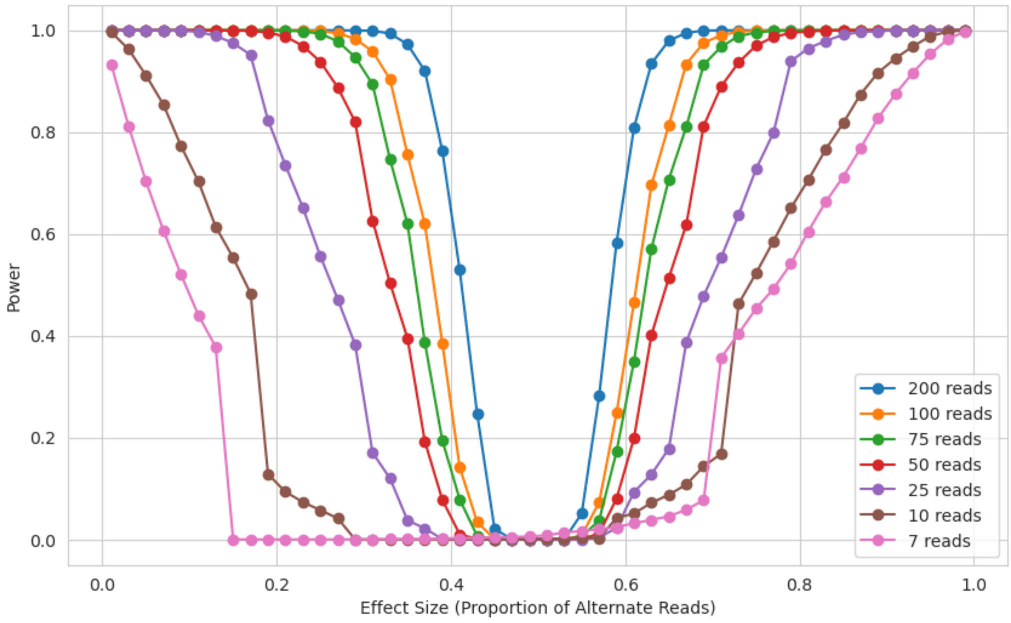

Figure SD3.1: Influence of effect sizes and read depth in successful ASE estimation after incorporating multiple tests.

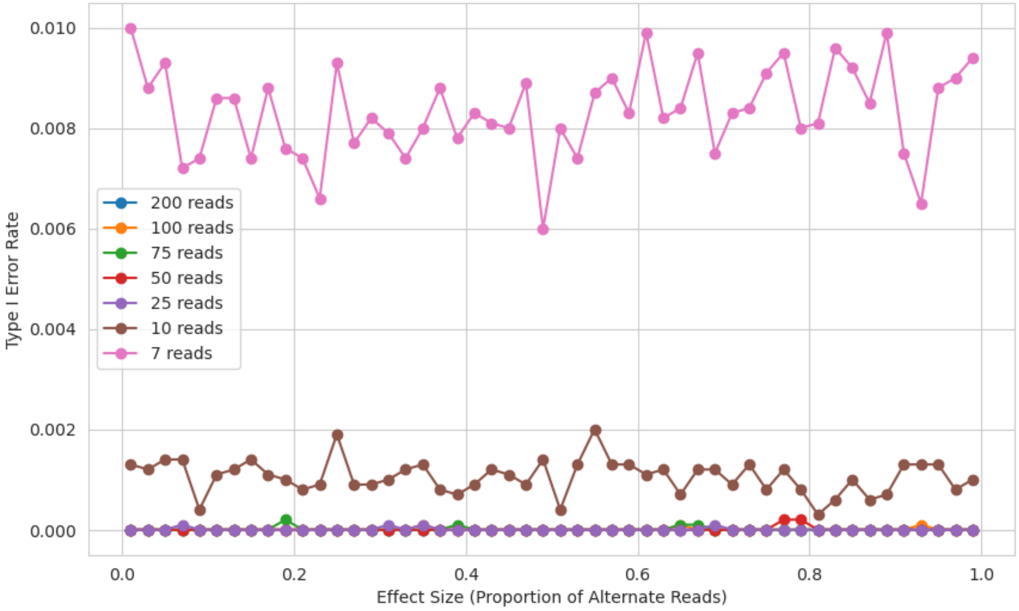

Figure SD3.2: Influence of effect sizes and read depth in Type-I error estimation.

4. Concordance between MBASED and SNV level beta-binomial test

MBASED employs meta-analysis, aggregating information across multiple SNVs to detect ASE at the gene level. In addition to MBASED, we used the beta-binomial test to identify SNV-level ASE, which is essential for understanding the specific effects of individual SNVs. We anticipated a certain degree of concordance between these two tests. Of the 730,673 genes assayed across 125 samples, 74.2% were not significant in either test. Conversely, 13.2% were deemed significant by both tests. Interestingly, 7.5% were significant only in MBASED, while 4.8% reached significance only in the beta-binomial test. It is important to note that a gene was classified as significant in the beta-binomial test if at least one heterozygous SNV located within that gene showed significance.

To gain a deeper understanding of the observed concordance and discrepancies between these two tests, we calculated and compared various metrics.

Deciles	0.1	0.2	0.3	0.4	0.5	0.6	0.7	0.8	0.9
Both	0.000	0.000	0.000	0.000	0.000	0.000	0.001	0.006	0.020
Neither	0.155	0.278	0.405	0.524	0.636	0.750	0.832	0.925	0.996
Beta Binom	0.078	0.118	0.174	0.249	0.346	0.465	0.602	0.757	0.909
MBASED	0.000	0.000	0.002	0.005	0.010	0.016	0.023	0.031	0.041

Table SD4.1: Distribution of MBASED P values across three groups.

Deciles	0.1	0.2	0.3	0.4	0.5	0.6	0.7	0.8	0.9
Both	0.000	0.000	0.000	0.000	0.001	0.004	0.008	0.019	0.033
Neither	0.164	0.263	0.379	0.493	0.591	0.725	0.837	0.931	1.000
Beta Binom	0.000	0.003	0.007	0.011	0.018	0.024	0.031	0.040	0.046
MBASED	0.069	0.084	0.111	0.147	0.173	0.230	0.278	0.401	0.630

Table SD4.2: Distribution of beta binomial P values across three groups.

Deciles	0.1	0.2	0.3	0.4	0.5	0.6	0.7	0.8	0.9
Both	0.15	0.25	0.33	0.50	0.56	0.80	1.00	1.00	1.00
Neither	0.00	0.00	0.00	0.00	0.00	0.00	0.00	0.00	0.00
Beta Binom	0.09	0.13	0.14	0.18	0.22	0.25	0.33	0.50	0.50
MBASED	0.00	0.00	0.00	0.00	0.00	0.00	0.00	0.00	0.00

Table SD4.3: Proportion of SNVs with beta-binomial p values < 0.05.

Deciles	0.1	0.2	0.3	0.4	0.5	0.6	0.7	0.8	0.9
Both	1	2	3	4	5	6	8	11	16
Neither	1	1	1	1	2	2	3	4	6
Beta Binom	2	3	4	4	5	7	8	10	14
MBASED	1	2	2	3	3	4	5	7	9

Table SD4.4: Number of SNVs in a gene

Deciles	0.1	0.2	0.3	0.4	0.5	0.6	0.7	0.8	0.9
Both	15	20	26	34.5	46	64	92.5	148	304
Neither	13	16	20	25	32	43	58.5	86	152
Beta Binom	17	22	27.5	35	44.5	57.5	77	111	195
MBASED	14	17	22	27	35	46	63.5	95	170

Table SD4.5: Total read number of reads

Deciles	0.1	0.2	0.3	0.4	0.5	0.6	0.7	0.8	0.9
Both	0.000	0.000	0.000	0.002	0.019	0.085	0.245	0.585	0.991
Neither	0.024	0.085	0.172	0.279	0.403	0.539	0.681	0.826	0.959
Beta Binom	0.000	0.000	0.000	0.001	0.003	0.008	0.017	0.038	0.091
MBASED	0.000	0.015	0.092	0.228	0.384	0.548	0.709	0.864	0.985

Table SD4.6: Median heterogeneity among SNVs in a gene identified by MBASED.

MBASED demonstrates increased power to detect subtle ASE, especially in genes with a greater number of SNVs, even when the allelic imbalance is not significant at every individual SNV. This is reflected in a higher proportion of significant SNVs and lower MBASED p-values in genes significant only by MBASED. Notably, MBASED appears tolerant of heterogeneity in ASE signals among SNVs within a gene, as evidenced by higher heterogeneity p-values in genes uniquely identified by MBASED. Conversely, genes significant only in the beta-binomial analysis often exhibit a more mixed heterogeneity pattern, potentially explaining why MBASED might not flag them as having consistent gene-level ASE. These findings underscore the importance of considering both the number of informative SNVs, the consistency of the ASE signal across those SNVs, and the inherent differences in the statistical frameworks of the two methods when interpreting results.

5. Highly expressed genes more often show ASE

We examined the relationship between gene expression levels (TPM) and ASE using a mixed-effects logistic regression model. Using gene expression (TPM) as a fixed effect and individual

sample names as a random effect, the analysis revealed that with a rise in gene expression, there is an increase in the log odds (with a coefficient of 1.96) of a gene exhibiting ASE. This aligns with the prior reports indicating that highly expressed genes more frequently exhibit ASE, presumably due to greater selection pressures. Genes with higher expression levels have been shown to be more efficient at suppressing the expression of deleterious alleles.
